# Enhanced osteoinductivity and corrosion resistance of dopamine/gelatin/rhBMP-2–coated β-TCP/Mg-Zn orthopedic implants: An *in vitro* and *in vivo* study

**DOI:** 10.1371/journal.pone.0228247

**Published:** 2020-01-30

**Authors:** Congcong Liu, Jingcheng Wang, Chengde Gao, Zhenting Wang, Xiaohua Zhou, Mingying Tang, Kun Yu, Youwen Deng

**Affiliations:** 1 Department of Orthopedics, The Second Xiangya Hospital, Central South University, Changsha, Hunan, P.R. China; 2 Department of Emergency Medicine, The Second Xiangya Hospital, Central South University, Changsha, Hunan, P.R. China; 3 State Key Laboratory of High Performance Complex Manufacturing, College of Mechanical and Electrical Engineering, Central South University, Changsha, Hunan, P.R. China; 4 Department of Spine Surgery, The Second Xiangya Hospital, Central South University, Changsha, Hunan, P.R. China; 5 School of Materials Science and Engineering, Central South University, Changsha, Hunan, P.R. China; University of South Carolina, UNITED STATES

## Abstract

Magnesium-based biomaterials are attracting increasingly more attention for orthopedic applications based on their appropriate mechanical properties, biodegradability, and favorable biocompatibility. However, the high corrosion rate of these materials remains to be addressed. In this study, porous β-Ca_3_(PO_4_)_2_/Mg-Zn (β-TCP/Mg-Zn) composites were fabricated via a powder metallurgy method. The β-TCP/Mg-Zn composites with 6% porosity exhibited optimal mechanical properties, and thus, they were selected for surface modification. A novel dopamine/gelatin/recombinant human bone morphogenetic protein-2 (rhBMP-2) coating with demonstrated stability was prepared to further improve the corrosion resistance of the composite and enhance early osteoinductivity. The homogeneously coated β-TCP/Mg-Zn composite showed significantly improved corrosion resistance according to electrochemical and immersion tests. In addition, extracts from the dopamine/gelatin/rhBMP-2–coated β-TCP/Mg-Zn composite not only facilitated cell proliferation but also significantly enhanced the osteogenic differentiation of Sprague–Dawley rat bone marrow-derived mesenchymal stem cells *in vitro*. Furthermore, *in vivo* experiments were performed to evaluate the biodegradation, histocompatibility, and osteoinductive potential of the coated composite. No obvious pathological changes in the vital visceral organs were observed after implantation, and radiography and hematoxylin-eosin staining showed strong promotion of new bone formation, matched composite degradation and bone regeneration rates, and complete absorption of the released hydrogen gas. Collectively, these results indicate that the dopamine/gelatin/rhBMP-2–coated β-TCP/Mg-Zn composite offers improved corrosion resistance, favorable biocompatibility, and enhanced osteoinductive potential for use in the fabrication of orthopedic implants.

## Introduction

Magnesium (Mg) is considered to have potential as a new generation of orthopedic metallic material [1], based on an elastic modulus comparable to that of natural bone [2–4], favorable biodegradability [5,6], and strong ability to promote new bone formation [4,7]. However, Mg corrodes easily both *in vitro* and *in vivo* due to a relatively low standard electrode potential [5]. Rapid corrosion leads to premature loss of mechanical integrity [6], an alkaline local environment [8,9], and a massive release of degradation by-products (Mg^2+^, hydroxyl ion, and hydrogen gas) [10–13], which further cause implant failure and induce adverse effects. Therefore, strategies to decrease the corrosion rate to better match the bone healing rate have become a research focus [14]. To delay corrosion, the composition of Mg-based materials was first optimized [1]. Al, Mn, Ca, Zn, and specific rare earth elements were used as alloying elements to improve the corrosion resistance and mechanical properties of Mg-based materials [15–18]. Our previous study [19] showed that addition of the appropriate amounts of Zn and β-Ca_3_(PO_4_)_2_ (β-TCP) improved the mechanical properties and corrosion resistance of Mg-based materials. Although a porous structure is essential for promoting the ingrowth of new capillaries and integration of bone tissue [11], greater porosity further reduces the corrosion resistance of Mg-based materials [3].

Application of a protective coating has been proven to be an effective method for decreasing the corrosion rate of a material, as it reduces the contact area between the substrate and a corrosive medium [20,21]. Previously reported coatings that not only improved the corrosion resistance but also enhanced the biocompatibility of biomaterials included Ca-P coatings [22–24], oxide coatings [25–27], fluoride coatings [28–30], and biodegradable polymer coatings [31,32]. Furthermore, the construction of composite coatings has been proposed in recent years [33,34]. Our previous study [35] revealed that application of composite dopamine/gelatin coatings decreased the corrosion rate of Mg-based materials. However, surface coating of Mg-based materials inevitably hinders the release of magnesium ions in the early stage after implantation, which negatively affects the promotion of osteogenesis. To overcome this drawback, early osteogenesis can be promoted by loading the coating with osteoinductive growth factors. As one of the most important growth factors in bone tissue engineering research, recombinant human bone morphogenetic protein-2 (rhBMP-2) has been demonstrated to effectively induce osteoblastic differentiation of cells of various types [36,37]. However, rhBMP-2 easily diffuses *in vivo*, making it difficult to promote local osteogenesis with long-lasting effects. Therefore, an appropriate carrier for rhBMP-2 is needed to achieve stable and sustained release. Xia et al [38] used gelatin microspheres to encapsulate BMP-2 on a poly(lactic-co-glycolic acid) scaffold and achieved sustained release. In addition, the coating also enhanced the proliferation and osteogenesis of rabbit bone mesenchymal stem cells (BMSCs) *in vitro*. Another study by Wang et al. focusing on the capacity for drug delivery showed that sustained BMP-2 release was obtained with both microspheres and nanosphere-based gelatin gels [39].

In the present study, to simultaneously improve the corrosion resistance of Mg based biomaterials and enhance the promotion of early osteogenesis, we loaded rhBMP-2 onto gelatin microspheres for sustained release. Additionally, polydopamine was used as an intermediate layer to promote adherence of the gelatin/rhBMP-2 layer onto the surface of porous 5%β-TCP/Mg-3%Zn composites, which were fabricated using powder metallurgy. Subsequently, the corrosion behavior, cytocompatibility, and osteoinductivity of the composites were tested *in vitro*. Furthermore, the *in vivo* biotoxicity, osteoinductivity, and biodegradation also were systematically evaluated.

## Materials and methods

### Material fabrication

The raw materials for the composites included pure Mg (purity ≥99.5%, particle diameter: 70–150 μm), pure Zn (purity ≥99.8%, particle diameter ≤45 μm), and β-Ca_3_(PO_4_)_2_ (β-TCP) particles (particle diameter ≤8 μm). Ammonium bicarbonate particles (NH_4_HCO_3_, purity ≥99.9%, 150 μm≤ particle diameter ≤400 μm) were utilized as space-holder particles. Powders of 92 wt.% Mg, 3 wt.% Zn and 5 wt.% β-TCP were mixed together in a vacuum tank for 24 h at room temperature. Then, NH_4_HCO_3_ particles were added to these mixed powders in 15%, 30%, and 45% volume percentages. The raw materials and space-holder particles were mixed for another 24 h. The mixtures were uniaxially compressed at 150 MPa (room temperature) into compact samples of 42 mm × 6 mm × 6 mm, 42 mm × 10 mm × 6 mm, and Φ20 mm × H20 mm using customized molds.

The sintering process for the compact samples included three steps. First, the compact samples were heated to 150–260 °C at a rate of 4 °C/min, and the temperature was maintained for 3 h in a vacuum furnace for removal of the space-holder particles. Second, the samples were heated to 550–610 °C under a high-purity argon atmosphere in a sinter furnace at a rate of 8 °C/min and incubated for 3 h. Finally, the sintered samples were cooled to room temperature under the protection of high-purity argon.

### Porosity measurements and microstructural characterization

The total porosities of 5%β-TCP/Mg-3%Zn composites were measured by gravimetry based on the following formula [40]: Porosity = (1-ρ/ρ_s_)×100%, where ρ_s_ represents the density of the 5%β-TCP/Mg-3%Zn specimens measured by the immersion method. The apparent density (ρ) of the 5%β-TCP/Mg-3%Zn specimens was measured by the weight-to-volume ratio.

The specimens were ground successively with 1000 and 1500 grit papers and polished with metallographic polishing paste and absolute ethanol. The surface morphology of the 5%β-TCP/Mg-3%Zn composites was observed under scanning electron microscopy (SEM; FEI Quanta-200, The Netherlands). Elemental analyses were conducted using energy-dispersive X-ray spectrometry (EDS; Phenom Pure, The Netherlands). Phase identification of the composites was performed by X-ray diffraction (XRD; Rigaku, DMAX-2500) using CuKα radiation at a wavelength of 1.5406 Å.

### Mechanical testing

The specimen size for the compression tests and Brinell hardness tests was Φ20 mm × H20 mm, and the specimen size for three-point bending tests was 42 mm × 6 mm × 6 mm. The compression and three-point bending tests were performed using a material test system (MTS810, USA). In addition, the hardness was tested with a Brinell sclerometer (HY, China). For three-point bending, the samples were placed on a holder with a 30-mm span to constitute a simple supported beam. The speed of the instrument was set at 1 mm/min for both compression tests and three-point bending tests. For all mechanical measurements, the data were collected until complete fracture of the specimen.

### Preparation and characterization of the dopamine/gelatin/rhBMP-2 coatings

Specimens with optimal mechanical properties were selected for coating modification. A dopamine-sodium hydroxide solution was prepared by mixing double distilled water, dopamine hydrochloride (Aladdin, China), and sodium hydroxide at a ratio of 1000:2:40. The 5%β-TCP/Mg-3%Zn composites were immersed in this solution for 72 h, followed by drying at room temperature and seal-preservation.

A gelatin solution was prepared using 50°C double distilled water and pharmaceutical-grade gelatin (Aladdin, China) in a ratio of 5:1. The gelatin solution was then dropped in preheated (50°C) liquid paraffin while stirring (500 r/min) for 3 min, followed by quick transfer of the suspension to a water bath (0 °C) with isopropanol while continuously stirring for 3 min. The suspension was then placed in a 4 °C refrigerator for 24 h for solidification, followed by the addition of a moderate amount of genipin and crosslinking for 12 h. The gelatin microspheres were dehydrated, dried, and sterilized by co-60 irradiation.

Gelatin microspheres (150 mg) were immersed in 5 ml rhBMP-2 solution (100 μg/mL). The microspheres were centrifuged and collected after shaking for 24 h on a 37 °C constant-temperature shaker. Each surface of the specimens was first brushed using a brush dipped in a gelatin solution and then dried in a biological safety cabin for 120 min. This process was repeated five times. Next, the rhBMP-2/gelatin microsphere was loaded. Finally, the microspheres were covered with gelatin solution five times.

### Electrochemical testing

The anti-corrosion properties were evaluated using potentiodynamic polarization testing by the MUL TI AUTOLAB M204 instrument while samples were immersed in Hank’s solution. Briefly, an Ag/AgCl electrode (saturated with KCl) and platinum plate were used as the reference and counter electrodes, respectively. The measurement was conducted after stabilizing the open circuit potential for 10 s. The scanning rate was 1 mV/s, the potential range was −2.0 V to 2.0 V, and the exposed area was 1 cm^2^. Triplicate tests were performed to ensure the repeatability of the results.

### In vitro immersion testing

The immersion tests, including the weight-change test, pH-value test and hydrogen-release test, were performed in Hank’s solution at 37 °C. All the specimens were ground with 1500 grit paper. The weight-change test and the pH-value test were performed using specimens of Φ20 mm × H20 mm size. The ratio of the solution volume and the specimen surface area was 20 ml/cm^2^. At each monitoring time point, the specimens were removed and dried for measurement of weight with an electronic balance (PL203, Mettler Toledo) and pH value using a benchtop pH meter (FE20, Mettler Toledo).

The hydrogen-release test was conducted using specimens of 10 mm × 10 mm × 6 mm size embedded in a denture base resin. The hydrogen generated by the immersed specimens was collected into acid burets, and the amount of released hydrogen was measured according to the difference in the liquid level heights.

### In vitro cytology assays

#### Preparation of material extracts

The extracts of the bare and coated 5%β-TCP/Mg-3%Zn composites were prepared based on the International Organization for Standardization (ISO 10993–5) [41]. The ratio of the surface area to the culture medium was 2.5 cm^2^/ml. The bare and coated specimens were incubated for 72 h at 37 °C in Dulbecco’s Modified Eagle’s Medium: nutrient mixture F-12 (DMEM/F-12, Gibco). The original extracts were collected, and the pH value was adjusted to neutral. After filter sterilization, the extracts were supplemented with 1% penicillin/streptomycin (HyClone, USA) and 10% fetal bovine serum (FBS, Gibco, USA).

#### Cell preparation and culture

Sprague–Dawley rBMSCs were provided by Cyagen Biosciences Inc [42]. DMEM/F-12 supplemented with 10% FBS and 1% penicillin/streptomycin was used as the culture medium, and the rBMSCs were cultured at 37 °C in 5% CO_2_. The culture medium was exchanged every 72 h. A subculture of the cells was performed when the rBMSCs reached approximately 90% confluence. Cells in the third to sixth passage were used for these experiments.

#### Cell viability

The Cell Counting Kit-8 viability assay (CCK-8, Dojindo, Japan) was performed to evaluate the influence of the specimen extracts on rBMSC proliferation. Then, 100 μl/well of cell suspension (3×10^4^ cell/ml) were seeded into four 96-well plates (3599, Costar). After pre-cultivation at 37 °C with 5% CO_2_ for 24 h, the culture medium for the two experimental groups was replaced with the solution of the extracts from bare specimens and coated specimens. Four 96-well plates were incubated in the incubator (Forma3111, Thermo) for 24, 48, 72, or 96 h. Then, 10 μl CCK-8 solution was added to each well at each time point, followed by incubation for 2 h. The absorbance of each well was measured with a microplate absorbance reader (MK3, Thermo) at 450 nm. Five parallel samples were used for each experimental condition.

#### Alkaline phosphatase (ALP) activity and ALP staining assay

An ALP activity kit (21101ES60, Yeasen) was utilized to assess the ALP activity of cells exposed to the specimen extracts. First, 3×10^5^ rBMSCs were cultured in the prepared solutions of extract from bare or coated specimens in 6-well plates. After induction for 7 and 14 days, 10 μL of 10 mM p-nitrophenol standard solution was diluted with ALP assay buffer to 0.2 ml. After the obtained cell lysate was centrifuged, 50 μL supernatant, dilute p-nitrophenol standard solution, and 100 μL blank control were each added to each well in the 96-well plates. After incubation at 37 °C for 30 min, the reaction was quenched by adding 100 μL stop solution to each well. The ALP activity was quantified based on the absorbance at a wavelength of 405 nm obtained using a microplate absorbance reader (MK3, Thermo). The results were normalized to the total protein content [43], and all assays were performed in triplicate.

ALP staining was also performed at day 7 and day 14 with an ALP stain kit (40749ES50, Yeasen). First, the rBMSCs were fixed by 4% paraformaldehyde for 1 min, followed by washing with tween-contained Tris buffered saline three times. Then, the cells were stained with a freshly prepared ALP staining solution in the dark for 20 min. Finally, the staining was stopped by removing the staining solution and washing with phosphate buffer saline. The results were observed and photographed using an inverted light microscope (AE2000, Motic).

#### Real-time quantitative polymerase chain reaction (RT-qPCR) analysis

The expression levels of osteoblast-related genes collagen I (COL I), osteocalcin (OCN), and osteopontin (OPN) were measured with quantitative real-time PCR. Briefly, rBMSCs were cultured in the prepared solutions of extract from bare or coated specimens at a density of 1×10^3^ cells/well for 7 or 14 days. Glyceraldehyde-3-phosphatedehydrogenase (GAPDH) was utilized as the housekeeping gene for normalization. Table 1 lists the forward and reverse primer sequences used in this experiment. Each 20 μl reaction solution consisted of 10 μL 2× SYBR Green qPCR Mix, 1 μL of each primer, 1.0 μL cDNA, and 7.0 μL sterile purified water. The experimental conditions of the real-time PCR were as follows: 95 °C for 3 min followed by 39 cycles at 95 °C for 10 s and 60 °C for 30 s. The differences between the target and control CT values for each sample were calculated as ΔCT: ΔCT = CT (target)–CT (control). The relative expression level for each gene was acquired by transforming the logarithmic values into absolute values using the 2^-ΔΔCT^ method. All the experiments were performed in triplicate.

**Table 1:** Primer sequences of osteoblast-related genes used for real-time quantitative PCR.

Gene	Direction	Sequence (5'-3')
OPN	Forward	AGGACAGCAACGGGAAGACC
	Reverse	CATCCGACCGCTCCGCACTA
OCN	Forward	GAGGGCAGTAAGGTGGTGAA
	Reverse	CCATAGATGCGCTTGTAGGC
COL 1	Forward	AGAATATGTATCACCAGACGCAGAA
	Reverse	GCACGGAAACTCCAGCTGAT

### In vivo evaluation

#### Animal model and postoperative observation

Specimens with superior corrosion resistance and biocompatibility in vitro were utilized to perform *in vivo* experiments. Twenty-four New Zealand rabbits (2.8–3.2 kg, 12 males and 12 females) were supplied by Laboratory Animal Center of The Second Xiangya Hospital (animal use permit No.: SYXK [Xiang] 2017–0002). The rabbits were randomly divided into a control group (6) and experimental group (18). Before the surgical procedure, anesthesia was applied by intravenous injection of 30 mg/kg pentobarbital sodium via an ear vein. The operation sites were carefully shaved and sterilized. A 2.5-cm longitudinal incision was made, and the skin and subcutaneous tissue were incised. The femoral shaft was exposed after blunt dissection of the muscle. A drilled hole with 3.0-mm diameter was carefully made in the middle and distal third of the right femur using an orthopedic drill. A Φ3 mm × H6 mm pin formed from coated 5%β-TCP/Mg-3%Zn composite was inserted into the drilled hole. The incision was sutured layer by layer. Intramuscular injection of 40U penicillin was applied immediately after surgery and every 24 h thereafter for 3 days to prevent infection. All rabbits were examined for general condition, appetite, lameness, condition of the wound, and formation of subcutaneous emphysema.

#### Serum biochemical testing, histological evaluation, and radiographic evaluation

Venous blood specimens from six rabbits in the experimental group were collected at six time points, including immediately preoperatively and 7, 14, 30, 60 and 90 days postoperatively. The serum concentrations of Mg^2+^, Zn^2+^, and Ca^2+^ ions as well as blood urea nitrogen (BUN), creatinine (Cr), alanine aminotransferase (ALT), and aspartate aminotransferase (AST) were measured by a biochemical analyzer (LABOSPECT 003, Hitachi).

Four animals were sacrificed by overdose of pentobarbital sodium at each observation point (4, 8 and 12 weeks after implantation). The heart, liver, kidney, and bone tissue surrounding the implants were harvested. These specimens were fixed in 4% paraformaldehyde for 48 h. In addition, the bone tissues were decalcified with 10% ethylene diamine tetraacetic acid for 30 days. The specimens were dehydrated with gradient ethyl alcohol solutions and embedded in paraffin. A Leica RM 2245 microtome was utilized to cut the tissue into sections of 5 μm thickness. The sections were stained sequentially with hematoxylin and eosin (H&E). Radiographs of the implantation area were obtained at 4, 8, and 12 weeks postoperatively for *in vivo* observation of the degradation process, release of hydrogen, and bone regeneration process.

### Statistical analysis

The quantitative results obtained in this study were expressed as the mean ± standard deviation. Statistical analyses were performed using SPSS 22.0 software (SPSS Inc., Chicago, IL, USA). One-way analysis of variance (ANOVA) followed by Student-Newman-Keuls post hoc tests were used to analyze the differences between groups. Statistical significance was defined by *p*<0.05.

## Results

### Characterization of the 5%β-TCP/Mg-3%Zn composite microstructure

**Fig 1:**
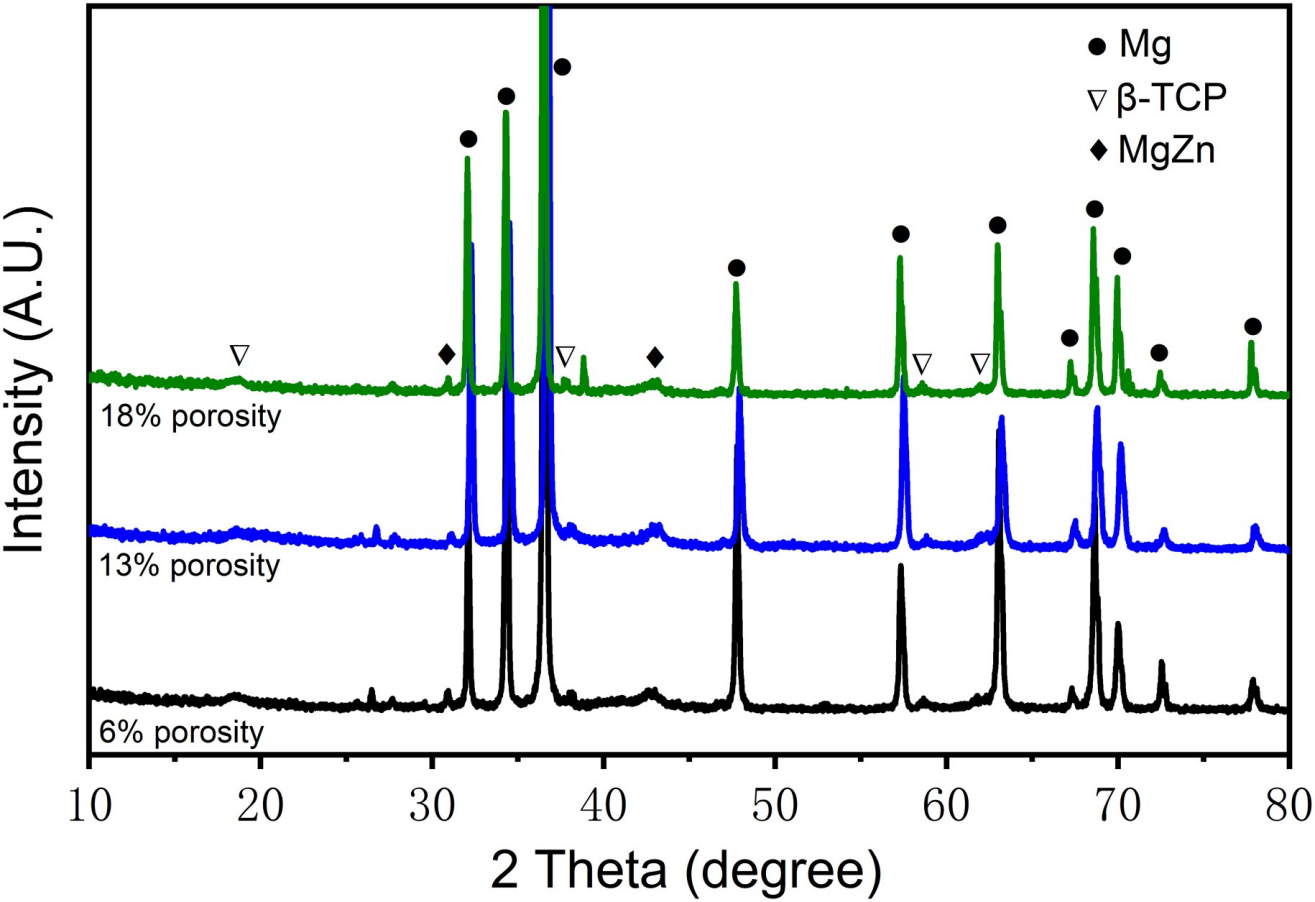
XRD patterns of the 5%β-TCP/Mg-3%Zn composites of different porosity.

The results of phase identification for the 5%β-TCP/Mg-3%Zn composites by XRD are shown in Fig 1. The α-Mg phases and Mg-Zn intermediate phases [2] were observed in all experimental specimens. In addition, the β-TCP phase was detected in the experimental specimens with 13% porosity. No ammonium bicarbonate phase, MgO phase, Mg-Ca phase or Zn-Ca phase was detected.

**Fig 2:**
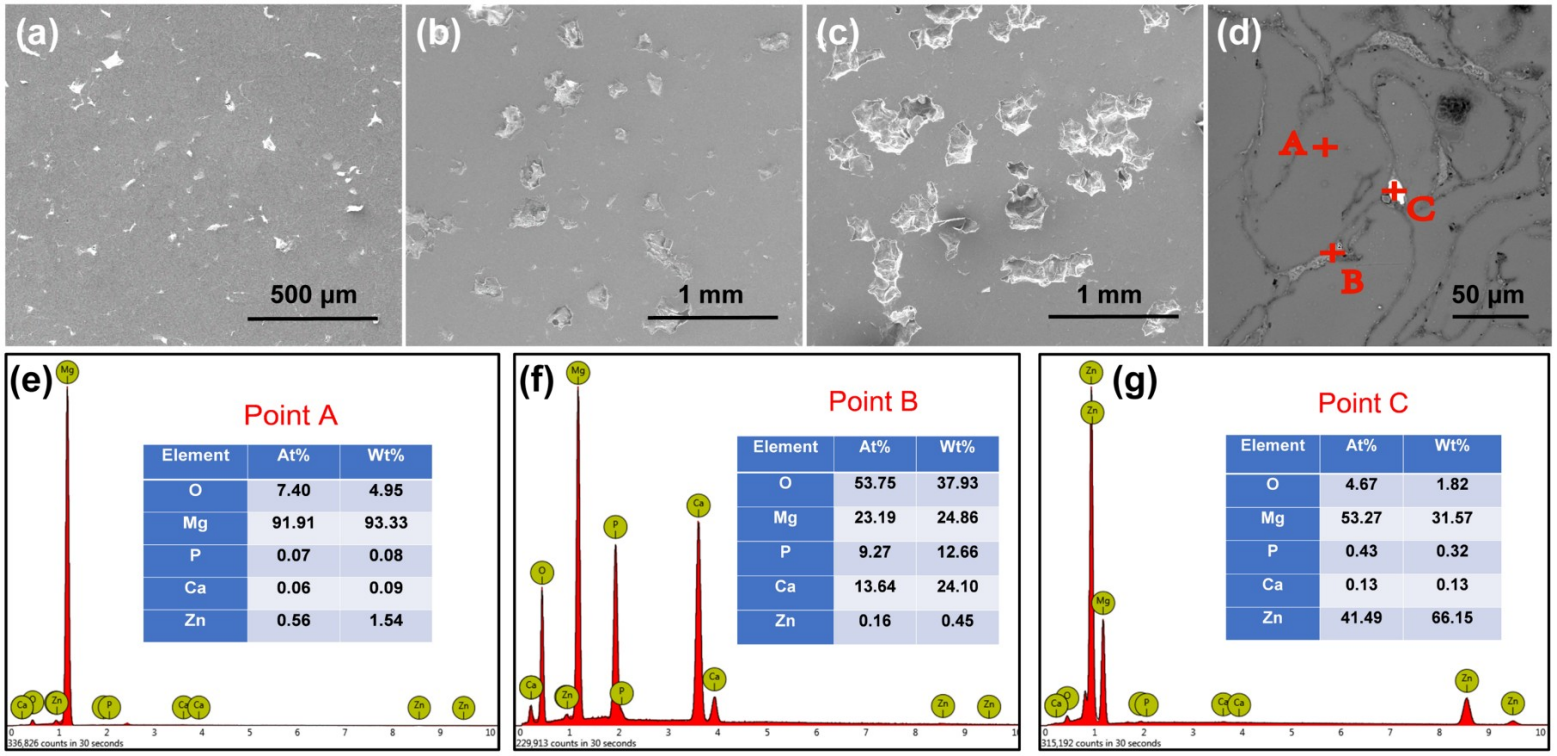
Surface morphologies (a-c) and elemental analysis (d-g) of the 5%β-TCP/Mg-3%Zn composites.

SEM micrographs and the results of EDS analysis of the 5%β-TCP/Mg-3%Zn composites are shown in Fig 2. A homogeneous pore distribution was observed. The average pore size and pore quantity increased with increasing space-holder content (Fig 2a–2c). The chemical composition of the grain area (point A) showed a large amount of Mg and little Zn; thus, it was identified as the α-Mg matrix (Fig 2e). The β-TCP phase (Fig 2f) was also identified along with the grain boundaries (point B), because the chemical composition showed a high quantity of Ca, P, and O. In addition, the Mg-Zn intermediate phases (Fig 2g) were observed in the grain boundaries (point C).

### Mechanical properties the 5%β-TCP/Mg-3%Zn composites

The porosities and mechanical properties of the porous 5%β-TCP/Mg-3%Zn composites in comparison with the porous Mg-6%Zn alloy and natural bone are summarized in Table 2. The mechanical properties of the porous 5%β-TCP/Mg-3%Zn composites decreased with increasing porosity. In addition, both the elastic modulus and Brinell hardness matched well those of natural bone. Compared with the porous Mg-6%Zn alloy, the addition of the appropriate ratio of β-TCP resulted in an increased compressive strength and a decreased elastic modulus. In addition, the compressive strength of the 5%β-TCP/Mg-3%Zn composite with 6% porosity was higher than that of natural bone.

**Table 2:** Porosity and mechanical properties of porous 5%β-TCP /Mg-3%Zn composites in comparison with porous Mg-6Zn and natural bone.

Material	Porosity (Vol.%)	UCS (Mpa)	UFS (Mpa)	F Modulus (Gpa)	Brinell Hardness
5%β-TCP /Mg-3%Zn composite	6	169.4	35.1	9.3	34.8
5%β-TCP /Mg-3%Zn composite	13	109.4	33.4	7.9	26.9
5%β-TCP /Mg-3%Zn composite	18	76.0	31.9	7.3	20.6
Porous Mg-6Zn [3]	6.7–17.3	49.5–95.5	44.6–66.7	8.3–11.1	-
Natural Bone [4,44,45]	-	1.8–150	2–180	0.1–20	20–58

UCS: uniaxial compressive strength; UFS: uniaxial flexural strength; F: flexural.

### Characterization of the dopamine/gelatin/rhBMP-2 coating applied to the 5%β-TCP /Mg-3%Zn composite

**Fig 3:**
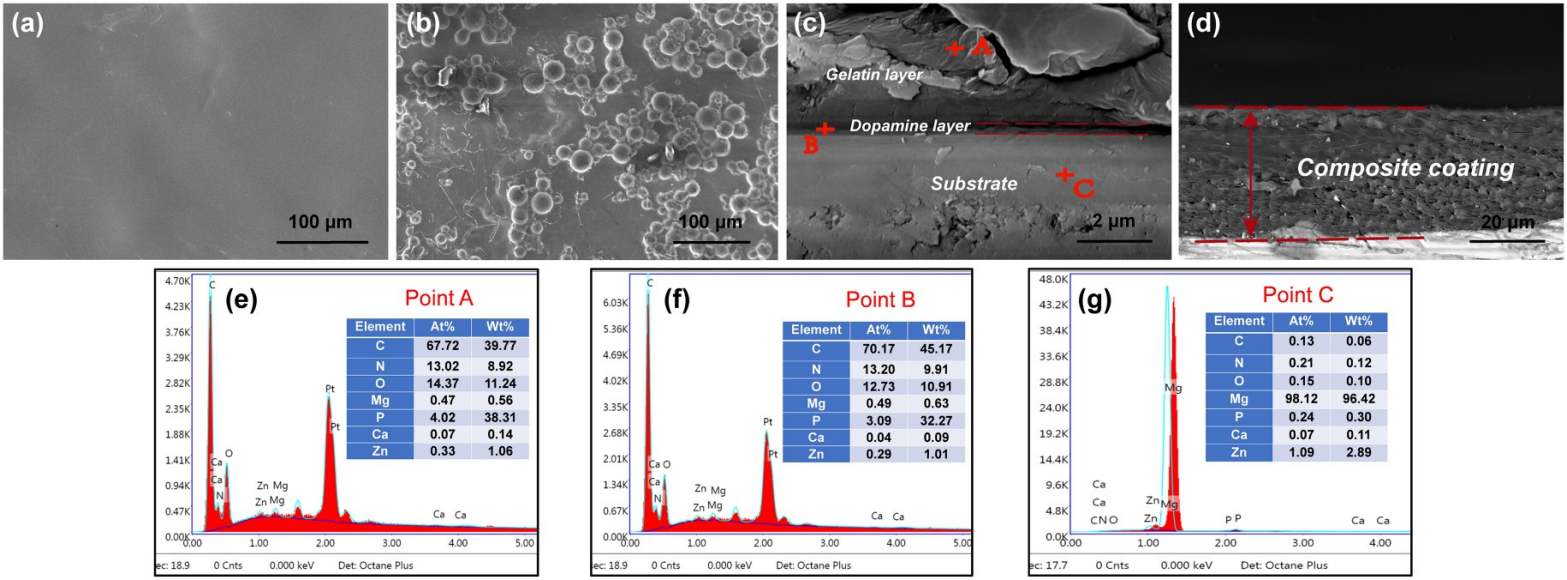
SEM images of the surface morphology of coated composites along with a cross-sectional view of the dopamine/gelatin/rhBMP-2 coating. (a) Gelatin surface; (b) BMP-2–loaded gelatin microparticles; (c) dopamine layer; and (d) the total composite coating. Elemental analysis of the (e) gelatin layer, (f) dopamine layer, and (g) substrate.

By SEM, the dopamine/gelatin/rhBMP-2 coating was observed to create a flat and smooth surface over the 5%β-TCP /Mg-3%Zn composite (Fig 3a). SEM images of the BMP-2–loaded gelatin microparticles used to prepare the coating showed the homogeneous distribution of the microparticles with intact sphere structures and no obvious aggregation (Fig 3b). From the cross-sectional view of the dopamine/gelatin/rhBMP-2 coating (Fig 3c and 3d), the dopamine layer was relatively thin, with an average thickness of 0.38±0.11 μm, and the thickness of the complete coating averaged 40.7±5.2 μm. In addition, no cracking was observed on the interphase between the substrate and the composite coating. EDS analysis of cross-sections of composite coating (point A) and the interphase between the substrate and the coating (point B) showed higher C, N and O content (Fig 3e and 3f), which indicated the presence of gelatin and dopamine.

### In vitro corrosion behavior of dopamine/gelatin/rhBMP-2-coated 5%β-TCP/Mg-3%Zn composite

**Fig 4:**
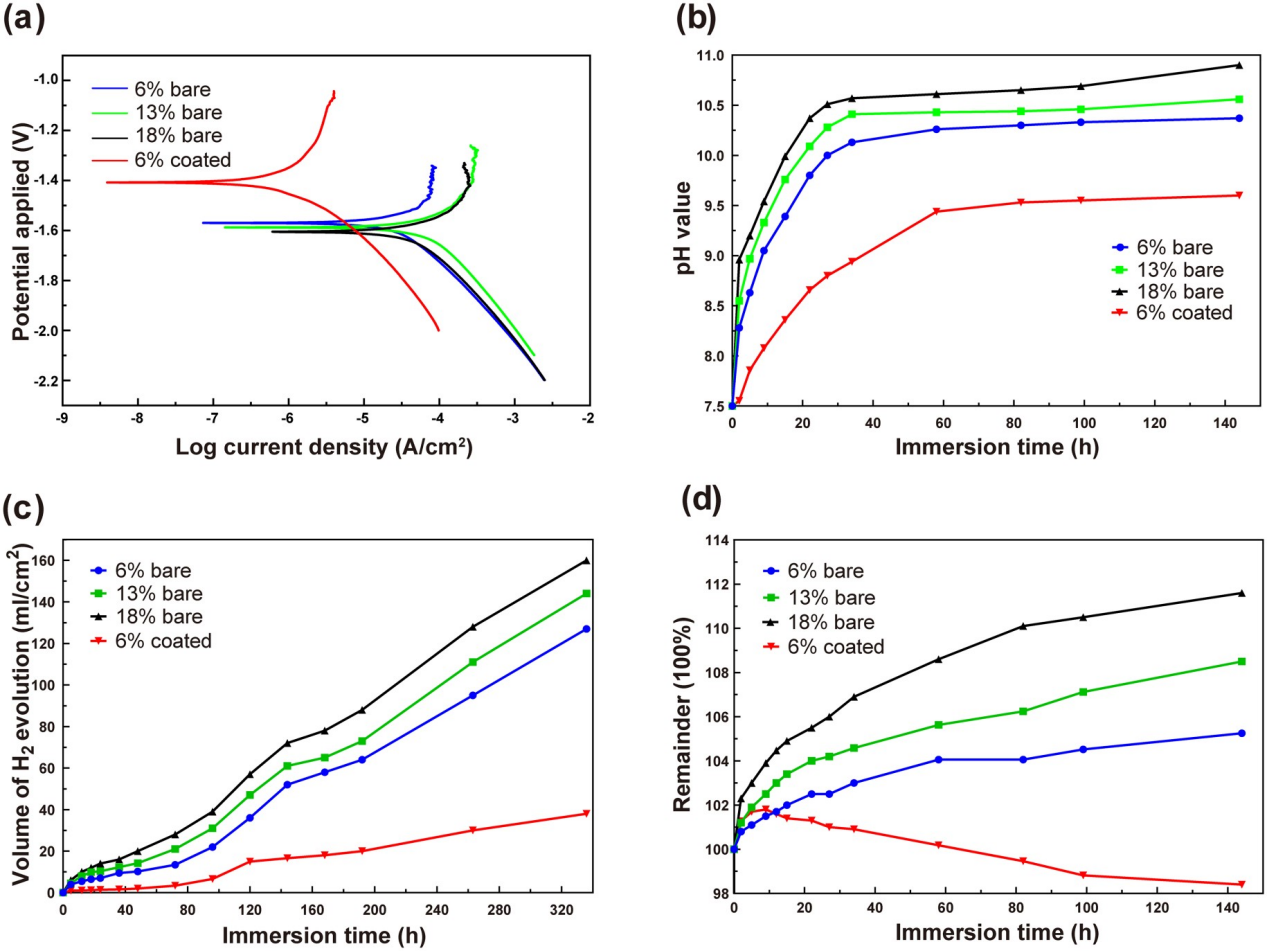
Anti-corrosion properties of dopamine/gelatin/rhBMP-2–coated 5%β-TCP/Mg-3%Zn composites. (a) Potentiodynamic polarization curves for the bare and coated 5%β-TCP/Mg-3%Zn specimens in Hank’s solution. (b) Variation in pH value of Hank’s solution with immersion time. (c) Volume of hydrogen produced during immersion. (d) Variation in weight of the immersed specimens.

Electrochemical tests were performed to evaluate the *in vitro* corrosion behavior of bare and dopamine/gelatin/rhBMP-2–coated 5%β-TCP/Mg-3%Zn composites. The self-corrosion potential (*E*_corr_) and current density (*i*_corr_) valued determined from extended Tafel slope analysis (Fig 4a) are presented in Table 3. For the bare specimens, with increasing porosity, the *E*_corr_ value decreased and the *i*_corr_ value increased. More importantly, the dopamine/gelatin/rhBMP-2–coated 5%β-TCP/Mg-3%Zn composite (6% coated) had the highest *E*_corr_ value (-1.40 V) and the lowest *i*_corr_ value (1.82 ×10^−6^ A/cm^2^). According to previous studies [46,47], enhanced electronegativity indicates a higher possibility of corrosion, whereas a lower current density suggests better corrosion resistance. Thus, the corrosion resistance of the different specimens can be ranked in the following order: 6% coated > 6% bare > 13% bare > 18% bare. Thus, a rationally selected porosity as well as coating with dopamine/gelatin/rhBMP-2 in this study endowed the best corrosion resistance to the composite.

**Table 3:** Corrosion potential (E_corr_) and corrosion current density (i_corr_) of all 5%β-TCP/Mg-3%Zn specimens in Hank’s solution.

5%β-TCP/Mg-3%Zn specimen	E_corr_(V)	i_corr_ (A/cm^2^)
Bare, 6% porosity	-1.57	4.26×10^−5^
Bare, 13% porosity	-1.59	7.76×10^−5^
Bare, 18% porosity	-1.61	9.55×10^−5^
Coated, 6% porosity	-1.40	1.82×10^−6^

Fig 4b shows the variation in the pH values of Hank’s solution surrounding the immersed specimens over time. For all bare specimens, the solution pH value increased rapidly during the initial 24 h. Then, the increase in the pH values slowed significantly and tended to stabilize in the late stage of immersion. Among the bare specimens, the specimens with 6% porosity produced the lowest pH values, which rose from 7.5 at the beginning to a maximum of 10.37 at 144 h, and specimens with 18% porosity produced the highest pH values, which rose from 7.5 to a maximum of 10.90 at 144 h. In addition, the pH values produced by coated specimens with 6% porosity were significantly lower than the pH values produced by any of the bare specimens, and the increase in pH value around the coated specimens was relatively slow and stable, reaching a value of 9.6 after 144 h.

The amount of hydrogen generated by all specimens was relatively small during the initial 48 h and gradually increased thereafter (Fig 4c). For bare specimens, the amount of hydrogen produced increased with increasing porosity (127 ml/cm^2^, 144 ml/cm^2^, and 160 ml/cm^2^ at 336 h for bare specimens with porosities of 6%, 13% and 18%, respectively). In addition, the coated specimens showed a much lower amount of hydrogen generation than the bare specimens, reaching a volume of only 38 ml/cm^2^ after 336 h.

The weight of all bare specimens increased gradually and slowly with prolonged immersion time (Fig 4d). Among the bare specimens, the specimens with 6% porosity had the minimum weight change of 105.25% over 144 h, while the specimens with 18% porosity had the maximum weight change of 111.6% over 144 h. For the coated specimens, the weight increased during the initial 9 h but began to decrease thereafter. In addition, the variability of the weight of the coated specimens was significantly smaller than that of the bare specimens with the same porosity, reaching 98.4% after 144 h. The weight curve reflected that the gelatin expanded with absorption of Hank’s solution at the beginning, followed by gradual peeling.

**Fig 5:**
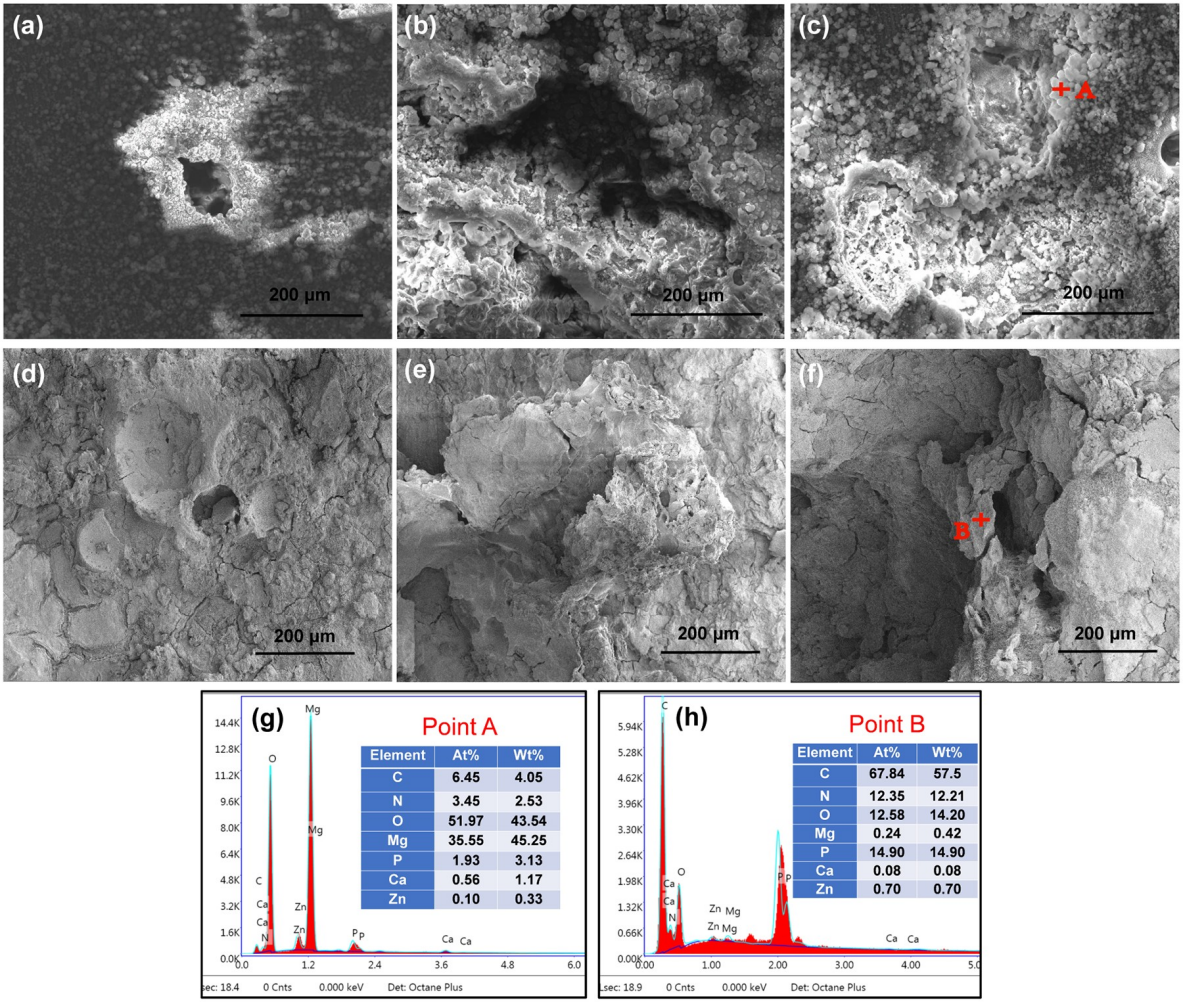
SEM images showing the surface morphologies of bare 5%β-TCP/Mg-3%Zn composites after immersion in Hank’s solution for 72 h: (a) 6% porosity; (b) 13% porosity; and (c) 18% porosity. SEM images showing the surface morphologies of dopamine/gelatin/rhBMP-2–coated 5%β-TCP/Mg-3%Zn composites after immersion in Hank’s solution for 5 h (d), 9 h (e) and 72 h (f). EDS analysis of the bare (g) and coated (h) specimen surfaces after immersion for 72 h.

SEM observation of the surface morphologies of the bare specimens after immersion in Hank’s solution for 72 h showed that corrosion initiated preferentially around the pores (Fig 5a–5c). The extent of corrosion and amount of corrosion products increased with increasing porosity. EDS analysis of the bare specimen surface showed higher O and Mg contents around the pores (Fig 5g), which indicated the formation of magnesium hydroxide. SEM observation of the surface morphologies of the coated specimens after 5, 9, and 72 h of immersion showed slight cracks beginning to appear on the surface of the coating after immersion for 5 h (Fig 5d). The gelatin layer showed considerable water absorption and expanded after immersion for 9 h (Fig 5e). Thereafter, a peeling phenomenon (Fig 5f) was observed for the gelatin. EDS analysis of the coated specimen surface showed higher C and O and N content (Fig 5h), which indicated the presence of a gelatin layer.

### Effects of 5%β-TCP/Mg-3%Zn composite extracts on rBMSC proliferation

**Fig 6:**
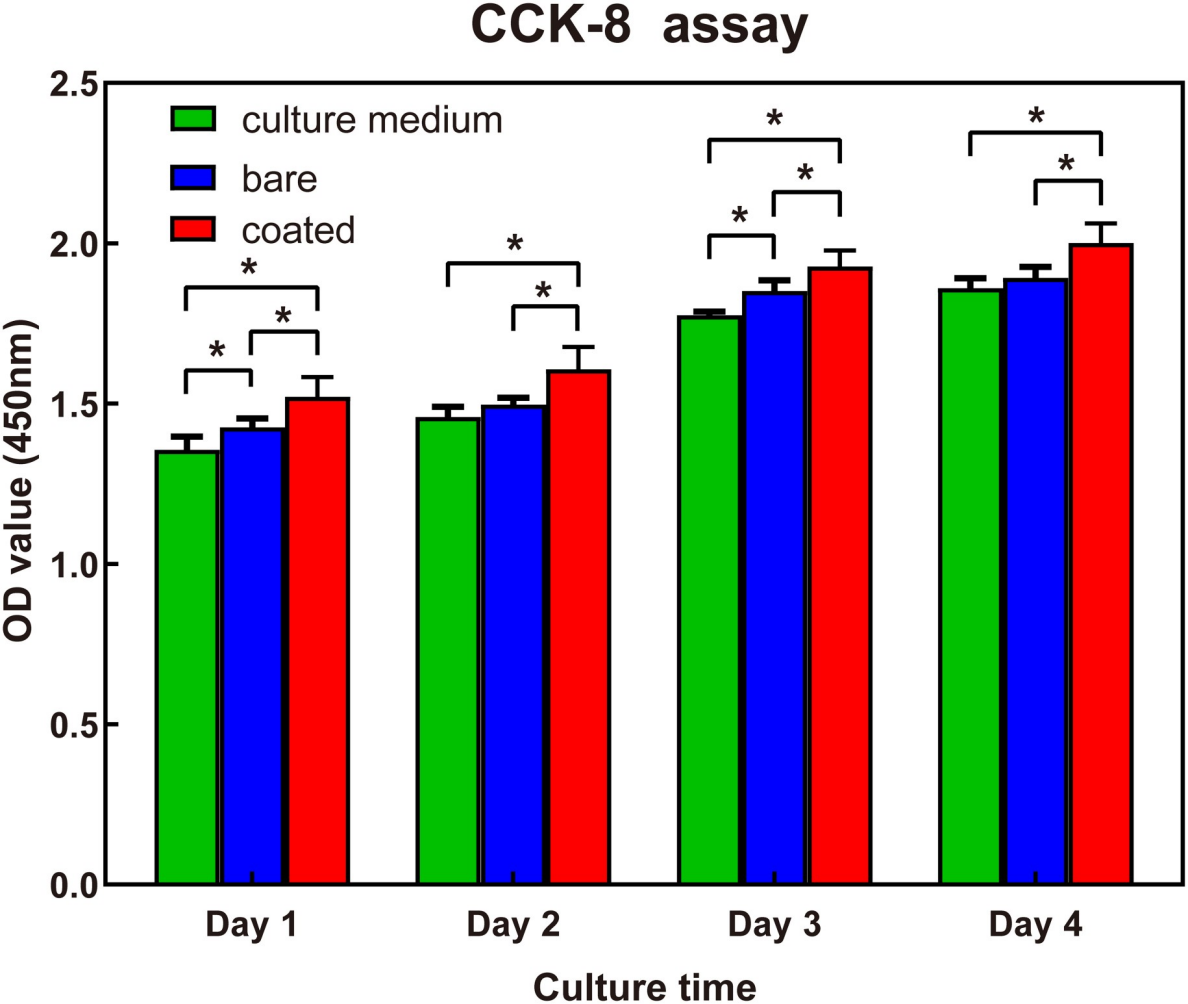
Cell viability of rBMSCs cultured with extracts from bare or coated 5%β-TCP/Mg-3%Zn composites over 4 days. *p<0.05.

The Mg^2+^ concentrations in the solutions of extract from the bare and coated 5%β-TCP/Mg-3%Zn composites were 12.88 mM and 5.94 mM, respectively, and for use in cell culture experiments, the pH values of both composite extract solutions were neutralized to 7.4. As shown in Fig 6, significantly greater rBMSC proliferation was induced by the coated specimen extract than by the bare specimen extract or culture medium at each time point (*p*<0.05). rBMSC proliferation induced by the bare sample extracts was higher than that by culture medium at 1 and 3 days (*p*<0.05). However, at 2 and 4 days, no obvious differences rBMSC proliferation was observed between the bare sample extracts and culture medium samples. These results indicate that compared with the extracts of the bare composite, the extracts of the coated composite better promoted the proliferation of rBMSCs.

### Effects of 5%β-TCP/Mg-3%Zn composite extracts on osteogenic differentiation of rBMSCs

**Fig 7:**
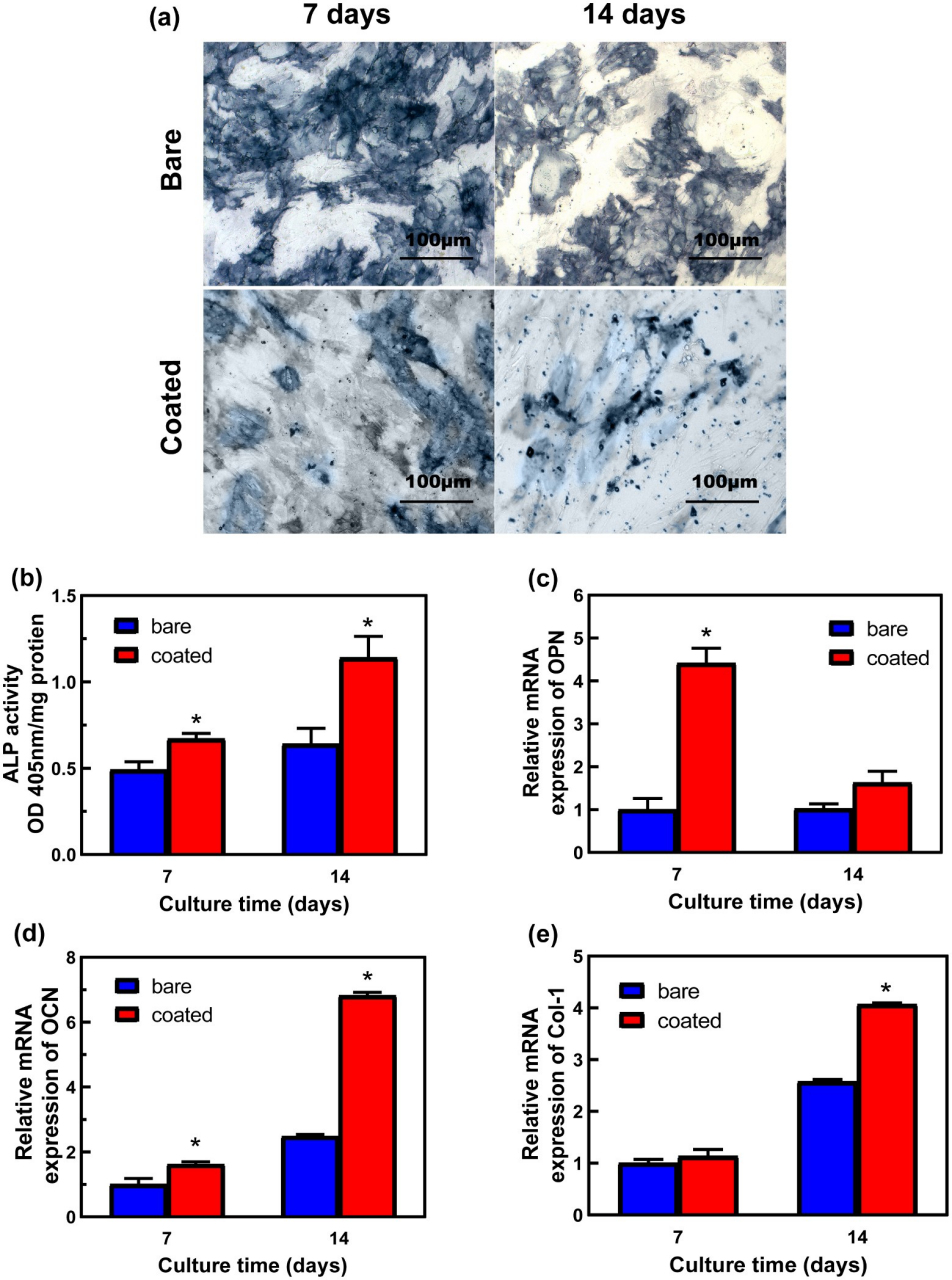
Osteogenic differentiation of rBMSCs cultured in solutions of extracts from bare or coated 5%β-TCP/Mg-3%Zn composites. (a) ALP staining after culture for 7 and 14 days; (b) quantitative ALP activity; (c-e) qPCR analysis of COL I, OCN, and OPN gene expression on days 7 and 14. *p<0.05.

Staining for ALP, a marker of osteogenic differentiation, was more intense among rBMSCs cultured in extract from the coated 5%β-TCP/Mg-3%Zn composite than among rBMSCs cultured in bare specimen extract on both days 7 and 14, with the most notable difference at day 14. Consistently, quantitative analysis revealed that the ALP activity in rBMSCs cultured with the coated composite extract was significantly higher than that in the rBMSCs cultured with bare sample extract on both days 7 and 14 (Fig 7b).

The relative mRNA expression levels of COL I, OCN, and OPN in rBMSCs cultured with the extracts of the bare or coated composites are shown in Fig 7c–7e. rBMSCs cultured with the coated composite extracts expressed significantly higher levels of OPN and OCN mRNA than cells cultured with the bare composite extracts on day 7 (Fig 7c and 7d). The OCN and COL I mRNA expression levels also differed significantly between rBMSCs cultured with the bare vs. coated composite extracts on day 14 (Fig 7d and 7e).

### In vivo behavior of dopamine/gelatin/rhBMP-2-coated 5%β-TCP/Mg-3%Zn composite following implantation in the rabbit femur

#### Serum biochemical evaluation and histopathological examination of vital visceral organs after composite implantation

**Fig 8:**
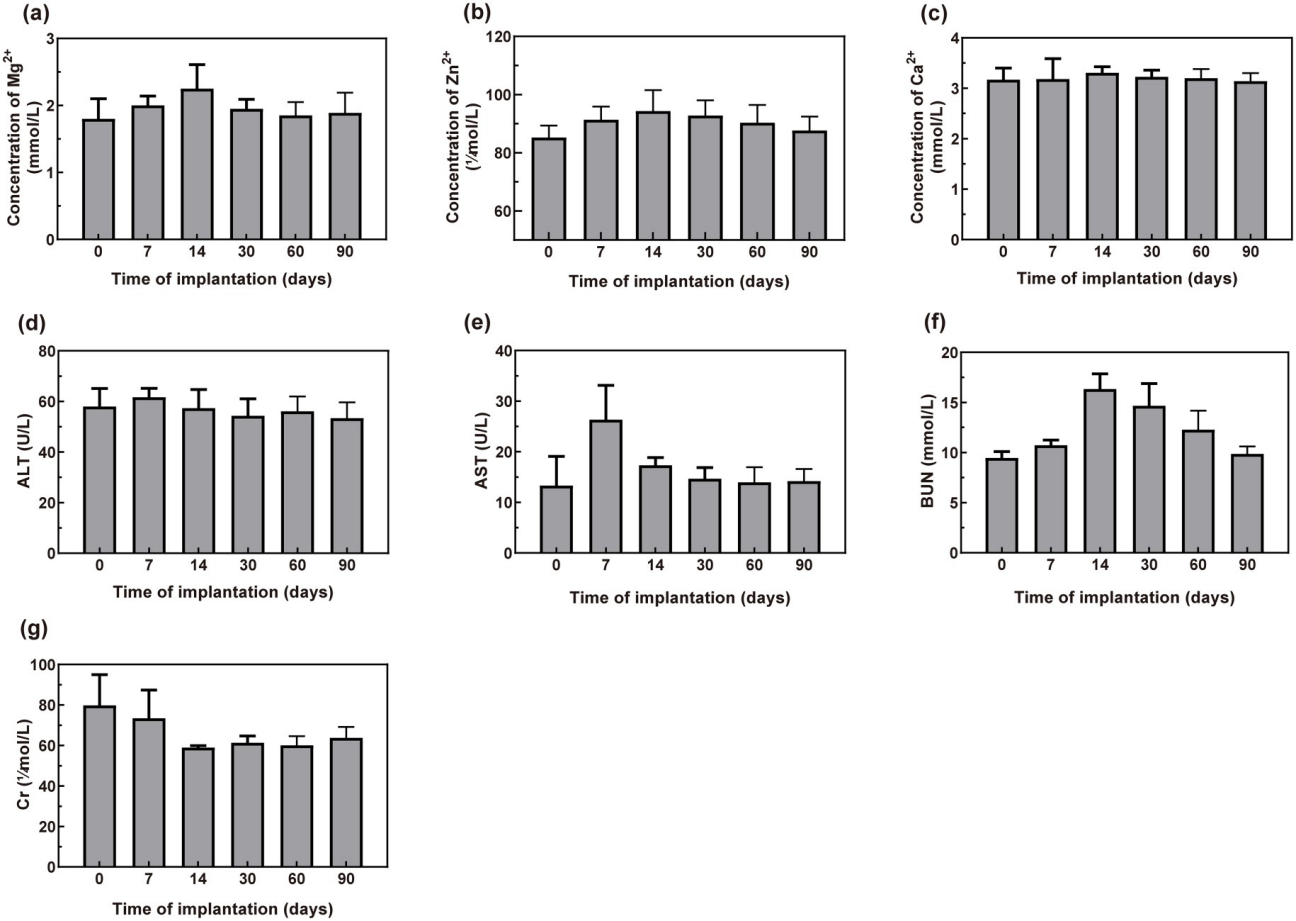
Serum concentrations of Mg^2+^ (a), Zn^2+^ (b), and Ca^2+^ (c) ions as well as ALT (d), AST (e), BUN (f) and Cr (g) in the experimental rabbits (n = 6) before and after implantation. Normal reference values for metal ions: Mg^2+^: 0.82–2.22 mmol/L, Zn^2+^:75.5–170 μmol/L, and Ca^2+^:3.1–5.2 mmol/L.

The serum concentrations of Mg^2+^, Zn^2+^, and Ca^2+^ ions as well as the major indicators of liver and kidney function in New Zealand rabbits were analyzed after implantation of a dopamine/gelatin/rhBMP-2-coated 5%β-TCP/Mg-3%Zn composite specimen in the rabbit femur (Fig 8). The serum concentration of Mg^2+^ increased to the maximum value (2.25±0.36 mmol/L) in approximately 14 days after implantation and then decreased gradually to the preoperative level (1.85±0.20 mmol/L) within approximately 60 days (Fig 8a). The concentrations of Zn^2+^ and Ca^2+^ reached peak values (94.31±7.2 μmol/L and 3.31±0.12 mmol/L, respectively) at 14 days after implantation, followed by continuous decreases to preoperative levels (87.6±4.8 μmol/L and 3.14±0.16 mmol/L, respectively), with very small ranges of variation (Fig 8b and 8c). The serum ALT level remained steady (from 61.70±3.50 U/L to 53.40±6.20 U/L), and the serum AST level fluctuated within the normal range (from 13.97±2.98 U/L to 26.33±6.80 U/L, Fig 8d and 8e). The BUN level showed a rapid increase up to 14 days postoperatively (16.33±1.53 mmol/L), followed by a slow decrease (Fig 8f). The serum Cr level decreased postoperatively (73.43±13.97 μmol/L) and tended to stabilize after 1 month (61.30±3.40 μmol/L, Fig 8g). These results indicate that the degradation of the implanted specimens did not result in abnormal fluctuations in serum ion concentrations or deficits in liver and kidney function.

**Fig 9:**
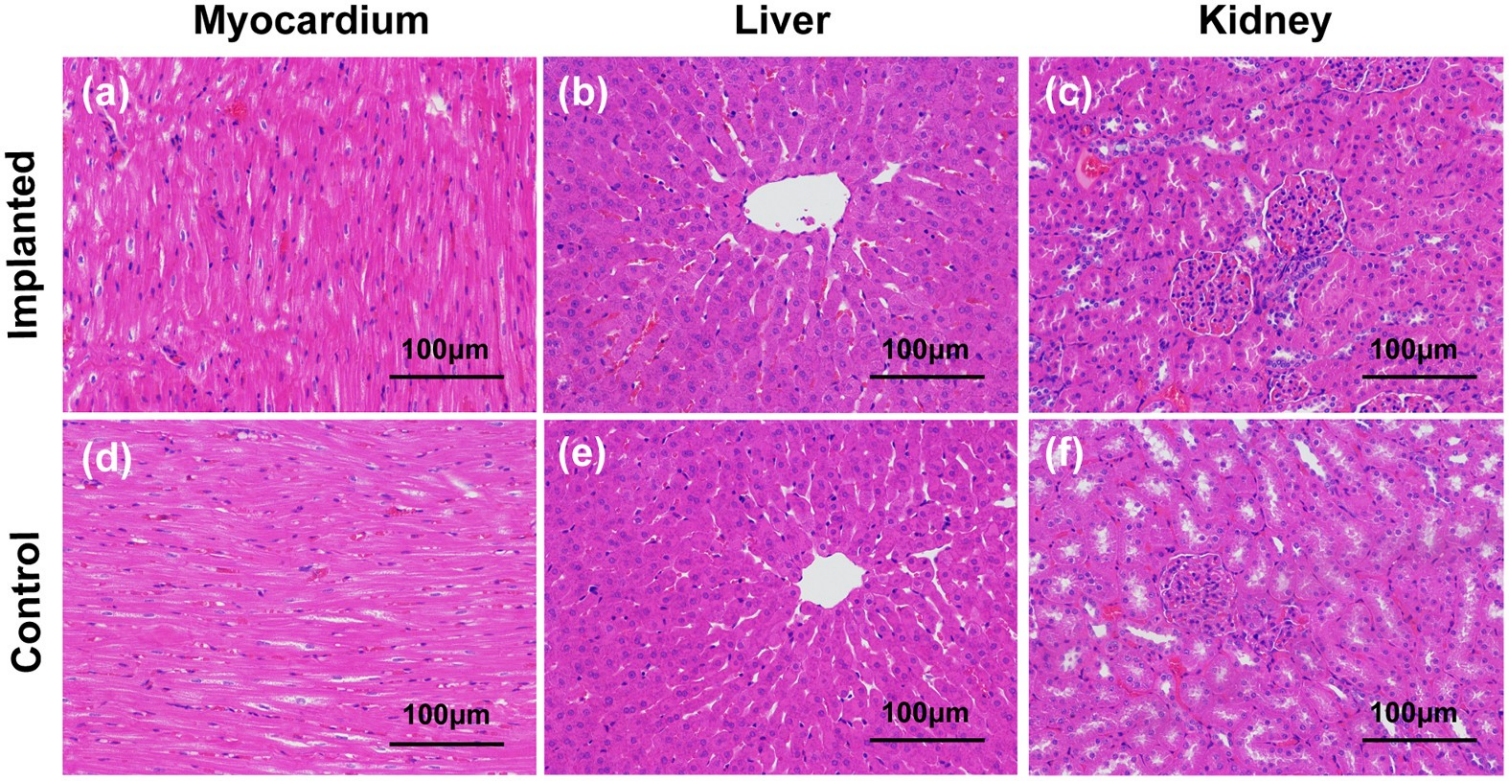
H&E staining of heart (a, d), liver (b, e) and kidney (c, f) tissue obtained from rabbits of the implantation and control groups at 1 month post-surgery.

Samples of rabbit heart, liver, and kidney harvested 1 month after coated composite implantation were analyzed histopathologically by H&E staining (Fig 9). No obvious necrosis or degeneration of the tissue was observed, indicating that the implanted specimens did not cause obvious damage to the vital visceral organs. These results were consistent with those of the blood biochemistry tests (Fig 8d–8g), and thus, satisfactory *in vivo* histocompatibility of the coated composite was demonstrated.

#### Radiographic examination of rabbit femurs after implantation of coated composite specimens

**Fig 10:**
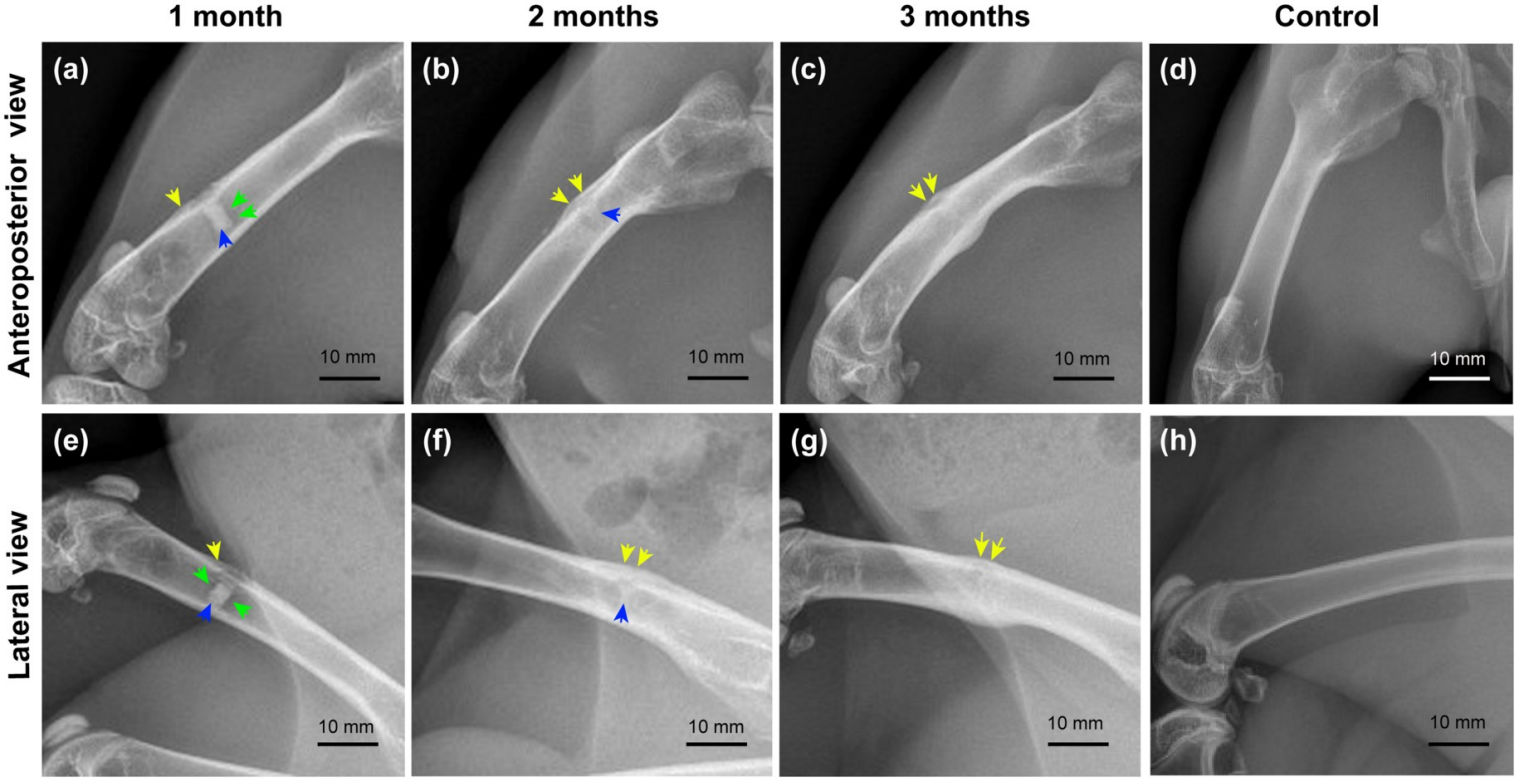
Radiographic evaluation of the implant site in the femur of New Zealand rabbits: (a-c) anteroposterior view of femurs in the experimental group; (e-g) lateral view of femurs in the experimental group; (d, h) anteroposterior and lateral view of femurs in the control group. Yellow arrows: periosteal reaction/callus; green arrows: hydrogen bubble; blue arrows: residual implant.

One month after implantation, anteroposterior and lateral radiographs of the femur showed a blurred edge of the coated composite implant (Fig 10a) and that the implant was surrounded by a low-density shadow (hydrogen bubble), which was relatively local (Fig 10e). In addition, a periosteal reaction could be observed (Fig 10a and 10e). These results suggest that corrosion of the implant had begun, and a small amount of hydrogen had been released. Two months after implantation, the shadow of the implant was smaller (Fig 10b and 10f) compared with that at 1 month after implantation (Fig 10a and 10e), and the gas bubble around it was barely visible. In addition, more and thicker calluses had formed in the bone defect area (Fig 10b and 10f). These phenomena indicated that the implant had further degraded, releasing hydrogen bubbles that had been absorbed, and that the bone defects were being repaired. Three months after implantation, the implant shadow was absent (Fig 10c), and the gas shadow had completely disappeared (Fig 10g). The newly regenerated cortex had rejoined at the defect hole. The cortex of the femur diaphysis where the drill hole was located was thickened (Fig 10c and 10g). These results suggested that degradation of the implant was complete by this time point, with thorough absorption of the hydrogen bubble and full repair of the bone defect.

#### Histological analysis of bone regeneration after coated composite implantation

**Fig 11:**
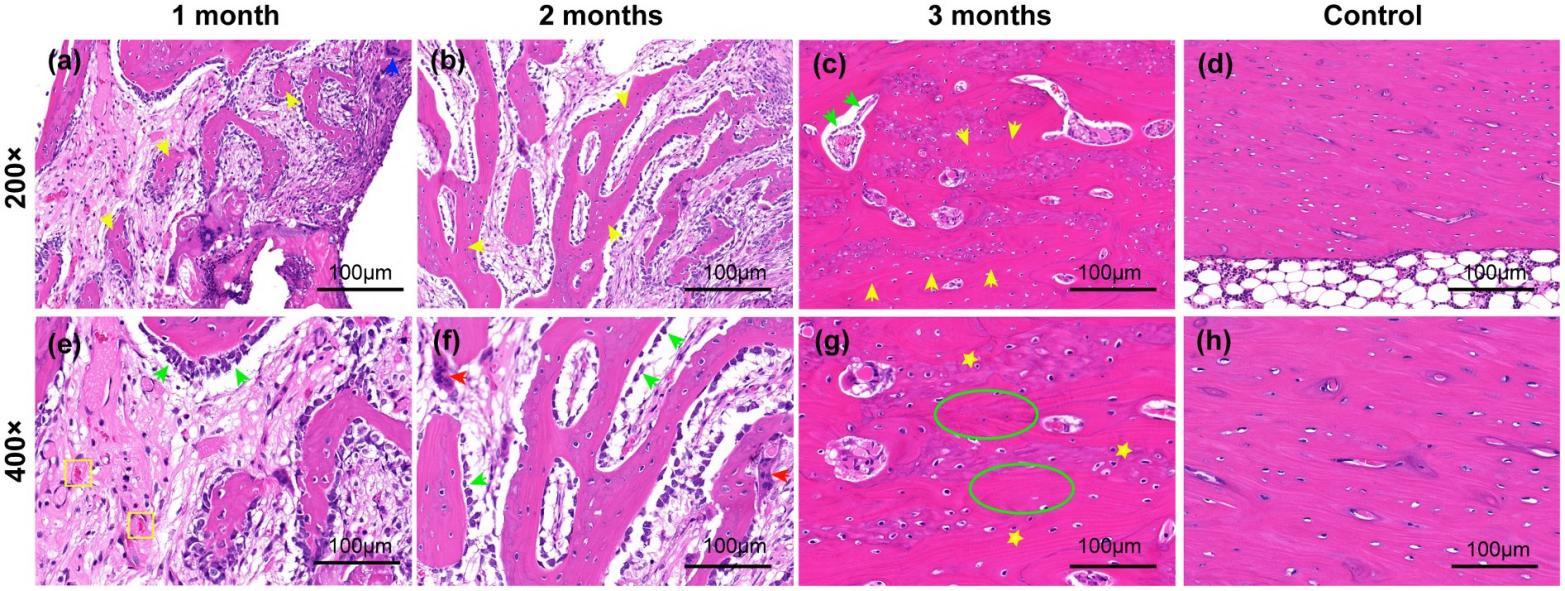
Representative images of H&E staining of decalcifying bone tissues around the implanted coated composite specimens at the indicated time points post-implantation. (a-c): Experimental group, 200×; (e-g): experimental group, 400×; (d, h) control group, 200× and 400×. Yellow arrows: newly formed trabeculae; blue arrows: multinucleated giant cells; green arrows: osteoblasts; yellow squares: new capillaries; red arrows: osteoclasts; yellow stars: osteocytes; green ellipses: lamellar bone matrix.

The process of bone regeneration after implantation of the coated 5%β-TCP/Mg-3%Zn composite was evaluated by histological staining (Fig 11). No obvious abnormality was detected in the bone tissues surrounding the implant at any time point, which implied favorable histocompatibility. At 1 month post-implantation (Fig 11a and 11e), new capillaries and newly formed trabeculae, which were surrounded by large amounts of continuously arranged osteoblasts, were observed. Isolated multinucleated giant cells (Fig 11e) were found in this field, presumably related to the degradation and absorption of implants. In addition, individual lymphocytes (Fig 11e) were observed, which suggested a postoperative aseptic inflammatory reaction. Two months after implantation (Fig 11b and 11f), more new trabeculae had formed with decreasing amounts of surrounding osteoblasts, and osteoclasts had also appeared (Fig 11f), which implied the gradual maturation of the newly formed bone. Predominantly mature bone tissue was observed in the defect area 3 months post-implantation (Fig 11c and 11g). Thickened trabeculae with sporadic osteoblasts (Fig 11g) was observed. In addition, the amount of the lamellar bone matrix had increased, and the size of the osteocytes decreased (Fig 11g), which indicated maturation of the bone tissue and essentially complete bone regeneration. These results were consistent with those of radiographic analysis.

## Discussion

Mg-based materials have been shown to be suitable orthopedic implant materials based on their adjustable mechanical properties, appropriate biodegradability, and favorable biocompatibility [19,48]. Based on our pilot study, adding 3 wt.% zinc and 5 wt.% β-TCP to the Mg substrate results in optimal mechanical and anti-corrosion properties. Furthermore, the mechanical properties of porous 5%β-TCP/Mg-3%Zn were shown to be closely correlated with the content of the space-holder particle [3]. The 5%β-TCP/Mg-3%Zn composite with 6% porosity exhibited optimal mechanical properties, having an enhanced compressive strength and a well-matched elastic modulus and Brinell hardness in comparison with natural bone (Table 2). Therefore, this composite composition was selected for coating with dopamine/gelatin/rhBMP-2 and further *in vitro* and *in vivo* studies.

According to previous studies, polydopamine (PDA) can be used as an intermediate layer for adhering silver [49], hydroxyapatite nanoparticles [50], heparin [51] or specific growth factors [52,53] onto the surface of biomedical metals. In this study, PDA successfully enhanced the bonding force between the gelatin/rhBMP-2 layer and the surface of the porous 5%β-TCP/Mg-3%Zn composite. The potential mechanism may involve the formation of a dopamine membrane with strong adhesion on the substrate surface [54–56] by self-polymerization under alkaline conditions.

Although surface coatings limit contact between the Mg substrate and corrosive medium, these protective layers inevitably hinder the release of Mg ions during the early stage of implantation, which can reduce the promotion of osteogenesis. Therefore, we introduced rhBMP-2 within the coating to improve the early osteoinductivity of the coated composite. However, rhBMP-2 easily diffuses *in vivo*, which makes long-term promotion of local osteogenesis difficult. Furthermore, locally high doses of rhBMP-2 can cause significant adverse reactions [57,58]. Gelatin microspheres have been demonstrated to be an ideal sustained-release carrier for rhBMP-2 in previous studies [38,59]. In addition, the low immunogenicity, good biocompatibility, and biodegradability of gelatin have been demonstrated [60]. Thus, gelatin microspheres were utilized to encapsulate rhBMP-2 to achieve sustained release from the composite coating. In the present study, a bioactive dopamine/gelatin/rhBMP-2 coating was successfully constructed on the surface of porous 5%β-TCP/Mg-3%Zn composite samples (Fig 3d). The catechol group from dopamine provides sufficient adhesive properties, so that the prepared coatings can adsorb onto the porous surface of the 5%β-TCP/Mg-3%Zn composite. Another special design was the rhBMP-2–loaded gelatin microspheres, which made the implants more biocompatible and stimulated osteogenic differentiation of BMSCs due to the sustained release of rhBMP-2. Consequently, the coated 5%β-TCP/Mg-3%Zn implants with improved corrosion resistance also showed favorable biocompatibility and enhanced osteoinductivity (Figs 4–7). In addition, these results were consistent with those of previous studies that used composite coatings containing PDA or gelatin [51,61,62].

Based on the decreased *i*_corr_ value and increased *E*_corr_ value, it is clear that the coated specimen with 6% porosity exhibited superior corrosion resistance compared with the bare specimens (with 6%, 13% or 18% porosity). In this study, increased porosity accelerated the degradation of the bare composites and reduced the anti-corrosion properties. A key design feature in this study was the introduction of the dopamine/gelatin/rhBMP-2 coating onto the 5%β-TCP/Mg-3%Zn composite, as this coating reduced the influence of the porous structure on the corrosion resistance of the Mg-based biomaterials. Our results demonstrate that selection of an appropriate porosity (6%) along with the protective effect of the coating improved the corrosion resistance of the dopamine/gelatin/rhBMP-2–coated 5%β-TCP/Mg-3%Zn composites.

According to a previous study [2], the addition of β-TCP in an appropriate proportion to Mg–Zn alloy significantly improves corrosion resistance. In addition, Ca_5_(PO_4_)_6_(OH)_2_ which is the reaction product of β-TCP and H_2_O, facilitates new bone formation [19]. The Mg matrix chemically reacts with simulated body fluid, producing hydroxides and releasing hydrogen.

$$\text{Mg}+2{\text{H}}_{2}\text{O}\to \text{Mg}{\left(\text{OH}\right)}_{2}+{\text{H}}_{2}↑$$ (1)

This reaction is the cause of the alkaline environment around the material (Fig 4b). According to this chemical reaction, Mg(OH)_2_ is the main solid corrosion product. Mg(OH)_2_ formed a protective layer on the surface of the composite specimen, preventing the corrosive medium from contacting the Mg matrix and, thus, delaying the corrosion process. In the present study, the results of the immersion test (Fig 4) showed that the corrosion resistance of the bare 5%β-TCP/Mg-3%Zn composites decreased with the increasing content of space-holder particles. Possible causes include the following: 1) the porosity and pore size increased with the increasing content of space-holder particles, resulting in an enlarged contact area between the composites and the corrosion medium, thereby accelerating corrosion [3,63]; and 2) a greater number of pores rapidly accommodated more chloride ions, promoting the occurrence of pitting corrosion [47,64]. Furthermore, in our immersion test, the presence of chloride ions transformed Mg(OH)_2_ into soluble MgCl_2_.

$$\text{Mg}{\left(\text{OH}\right)}_{2}+2{\text{Cl}}^{-}\to {\text{MgCl}}_{2}+2{\text{OH}}^{-}$$ (2)

The breakdown of this protective layer promotes the further corrosion of the substrate to a certain extent. The corrosion process stabilizes and slows when these two reactions [(1) and (2)] reach a dynamic equilibrium (Fig 4b), and these results were consistent with those of previous studies [3,19].

An indirect cytocompatibility test was performed in the present study using extract collected from the composite materials. According to a similar assessment [65], undiluted extracts from Mg alloys resulted in nearly complete cell death. However, this cytotoxicity is primarily attributed to high alkalinity and the condition of hyperosmosis, which are caused by nonphysiological degradation during the extraction process [66]. It is widely accepted that cell proliferation is inhibited in a hyperosmotic environment [67], whereas Mg^2+^ ions are actually nontoxic to cells [68,69]. In this study, owing to the protection of the composite coatings and the low solubility of magnesium hydroxide, Mg^2+^ ion concentrations in the extracts did not result in excessive osmotic pressure (5–20 mM Mg^2+^ ion can be tolerated by cells) [70,71]. Based on that information, non-diluted extract solutions adjusted to a neutral pH were utilized to perform the cytotoxicity assay in this study. As shown in Fig 6, the extract of the coated specimens that contained appropriate concentrations of Mg^2+^ ion and rhBMP-2 significantly promoted the proliferation of rBMSCs at each time point, compared with the extracts from the bare specimens, which is consistent with the results of previous studies [70,71]. Thus, our results indicate that the dopamine/gelatin/rhBMP-2 coatings not only prevent rapid release of Mg^2+^ but also improved the cytocompatibility of the 5%β-TCP/Mg-3%Zn composite.

The *in vitro* osteoinductive assays showed significantly higher ALP activity and upregulated expression of osteogenic genes in rBMSCs incubated in medium containing extract from the coated 5%β-TCP/Mg-3%Zn composite (Fig 7). The differences in the osteoinductivity of the bare and coated composite specimens may be due to the difference in Mg^2+^ ion concentration and the presence of rhBMP-2. In a study by Wong et al [72], osteogenic differentiation was promoted by a 50 ppm concentration of Mg^2+^ but inhibited by 200 ppm Mg^2+^, which indicated that the osteoinductivity of Mg^2+^ is concentration-dependent. From our measurement of Mg^2+^ concentrations produced by the bare and coated composites (12.88 mM and 5.94 mM Mg^2+^), we can conclude that these concentrations are capable of facilitating *in vitro* osteogenic differentiation of rBMSCs. In addition, the presence of rhBMP-2 in the extract significantly promoted the upregulation of osteoblast-related genes. However, there was no great disparity in the gene expression levels induced by the extracts from the bare vs. coated specimens. The reason may be that the concentration of rhBMP-2 in the extract was not very high due to the slow controlled release achieved by using the gelatin microspheres.

Yu et al. [19] implanted dense 10%β-TCP/Mg–6% Zn composites into the dorsal muscles and femoral shafts of Zelanian rabbits and found that the serum concentrations of Mg^2+^, Zn^2+^ and Ca^2+^ ions fluctuated within normal ranges. Consistently, the serum concentrations of Ca^2+^ and Zn^2+^ ions fluctuated within normal ranges in the present study after implantation of the coated composite (Fig 8b and 8c). Moreover, the concentration of Mg^2+^ ion did not reach the upper limit of the normal range (2.22 mmol/L) until 2 weeks after implantation (Fig 8a). This result indicated that the composite coating delayed the rapid release of Mg^2+^ ions that is caused by the initial rapid degradation. Prior research has shown that excess Mg^2+^ ions produced by degradation are mainly excreted via the urine to stabilize the serum concentration of Mg^2+^ ions [73]. Therefore, we also tested the serum levels of BUN, Cr, ALT and AST and performed histopathological analyses to evaluate the potential damage to the liver and kidney by the composite degradation products. The results showed that serum ALT and AST levels remained within the normal ranges, and although increased, the serum BUN concentration did not reach abnormal levels (Fig 8f). Thus, no clinically significant damage was observed. Tie et al. [74] observed the histopathological morphologies of the liver, spleen, and kidneys after implantation of a magnesium/strontium alloy and detected no remarkable histological differences between their test and sham groups. Consistently, no pathological changes were found in the harvested heart, liver and kidney tissues in the present study (Fig 9). Together, these results indicate that excess ions released during implant degradation can be removed from the body via normal metabolism, without causing definitive damage to the morphology or function of the vital viscera.

The rapid *in vivo* degradation of Mg-based materials has been an major limitation to their clinical use that needed to be addressed [75,76]. In the present study, the delayed *in vivo* degradation of coated porous 5%β-TCP/Mg-3%Zn composite was achieved by applying a dopamine/gelatin/rhBMP-2 coating. In our rabbit implantation model, the *in vivo* degradation rate of the coated composite was consistent with the bone regeneration rate, and the implant exhibited complete biodegradation (Fig 10a–10c and 10e–10g). In addition, the released hydrogen was absorbed thoroughly by 12 weeks after implantation (Fig 10c and 10g). This result is consistent with similar *in vivo* findings for Mg alloys [8]. The thorough absorption of hydrogen may be attributed to the limited size of the implant and the slow release of hydrogen due to the protective coating. Furthermore, the fully repaired bone defect observed by radiography (Fig 10c and 10g) and histological analysis (Fig 11) showed the gradual maturation of new bone tissue. No pathologic changes were detected in the bone tissue around the implants, which indicated the absence of local toxicity. Different amounts of macrophages and multinucleated giant cells were observed around the implants (Fig 11e), and these cells have been previously shown to act as scavenger cells that eliminate the products of corrosion [77,78]. The *in vivo* osteoinductivity of the dopamine/gelatin/rhBMP-2-coated 5%β-TCP/Mg-3%Zn composite may be attributed to the following factors: 1) the osteoinductivity of rhBMP-2 is strong in the early stage; 2) Mg^2+^ can promote the differentiation of BMSCs into osteoblasts [28] and promote the proliferation and migration of osteoblasts [79]; 3) phosphorus ions, calcium ions, and gelatin facilitate mineralization in new bone tissue; and 4) the three-dimensional porous structure is beneficial to the process of bone tissue infiltration.

## Conclusions

Porous 5%β-TCP/Mg-3%Zn composites coated with bioactive dopamine/gelatin/rhBMP-2 were successfully fabricated. The composites with 6% porosity exhibited superior compressive strength and a well-matched elastic modulus and Brinell hardness compared with natural bone. Moreover, the 5%β-TCP/Mg-3%Zn coated composites showed improved corrosion resistance and enhanced cytocompatibility and osteoinductivity, compared to those of the bare composites in vitro. The in vivo experiments further demonstrated the favorable histocompatibility of the coated composite with the local bone tissue and critical visceral organs of New Zealand rabbits. Furthermore, because the degradation rate of the composite was matched to the bone regeneration rate, we expect there to be enough space for new bone regeneration. Together our results indicate the dopamine/gelatin/rhBMP-2–coated 5%β-TCP/Mg-3%Zn composites offer improved corrosion resistance, favorable biocompatibility, and enhanced osteoinductivity, making this composite material an ideal candidate for use in the fabrication of orthopedic implants.

## References

[1] Agarwal S, Curtin J, Duffy B, Jaiswal S (2016) Biodegradable magnesium alloys for orthopaedic applications: A review on corrosion, biocompatibility and surface modifications. Mater Sci Eng C Mater Biol Appl 68: 948–963. 10.1016/j.msec.2016.06.020 27524097

[2] Yan Y, Kang Y, Li D, Yu K, Xiao T, et al (2017) Improvement of the mechanical properties and corrosion resistance of biodegradable beta-Ca3(PO4)2/Mg-Zn composites prepared by powder metallurgy: the adding beta-Ca3(PO4)2, hot extrusion and aging treatment. Mater Sci Eng C Mater Biol Appl 74: 582–596. 10.1016/j.msec.2016.12.132 28254333

[3] Yan Y, Kang Y, Li D, Yu K, Xiao T, et al (2018) Microstructure, Mechanical Properties and Corrosion Behavior of Porous Mg-6 wt.% Zn Scaffolds for Bone Tissue Engineering. Journal of Materials Engineering and Performance 27: 970–984.

[4] Staiger MP, Pietak AM, Huadmai J, Dias G (2006) Magnesium and its alloys as orthopedic biomaterials: a review. Biomaterials 27: 1728–1734. 10.1016/j.biomaterials.2005.10.003 16246414

[5] Song GL, Atrens A (1999) Corrosion Mechanisms of Magnesium Alloys. ADVANCED ENGINEERING MATERIALS 1: 11–33.

[6] Thormann U, Alt V, Heimann L, Gasquere C, Heiss C, et al (2015) The biocompatibility of degradable magnesium interference screws: an experimental study with sheep. Biomed Res Int 2015: 943603 10.1155/2015/943603 25717474 PMC4329844

[7] Zhang Y, Xu J, Ruan YC, Yu MK, O’Laughlin M, et al (2016) Implant-derived magnesium induces local neuronal production of CGRP to improve bone-fracture healing in rats. Nat Med 22: 1160–1169. 10.1038/nm.4162 27571347 PMC5293535

[8] Witte F, Kaese V, Haferkamp H, Switzer E, Meyer-Lindenberg A, et al (2005) In vivo corrosion of four magnesium alloys and the associated bone response. Biomaterials 26: 3557–3563. 10.1016/j.biomaterials.2004.09.049 15621246

[9] Edward G, Revie RW (2010) Corrosion Resistance of Aluminum and Magnesium Alloys: Understanding, Performance, and Testing: Wiley 719 p.

[10] Wang C, Shen J, Xie F, Duan B, Xie X (2017) A versatile dopamine-induced intermediate layer for polyether imides (PEI) deposition on magnesium to render robust and high inhibition performance. Corrosion Science 122: 32–40.

[11] Lai Y, Li Y, Cao H, Long J, Wang X, et al (2019) Osteogenic magnesium incorporated into PLGA/TCP porous scaffold by 3D printing for repairing challenging bone defect. Biomaterials 197: 207–219. 10.1016/j.biomaterials.2019.01.013 30660996

[12] Xu T, He X, Chen Z, He L, Lu M, et al (2019) Effect of magnesium particle fraction on osteoinduction of hydroxyapatite sphere-based scaffolds. J Mater Chem B 7: 5648–5660. 10.1039/c9tb01162e 31465084

[13] Höhn S, Virtanen S, Boccaccini AR (2019) Protein adsorption on magnesium and its alloys: A review. Applied Surface Science 464: 212–219.

[14] Horynová M, Remešová M, Klakurková L, Dvořák K, Ročňáková I, et al (2019) Design of tailored biodegradable implants: The effect of voltage on electrodeposited calcium phosphate coatings on pure magnesium. Journal of the American Ceramic Society 102: 123–135.

[15] Song GL, Atrens A, Wu XL, Zhang B (1998) Corrosion behaviour of AZ21, AZ501 and AZ91 in sodium chloride. Corrosion Science 40: 1769–1791.

[16] Ding Y, Wen C, Hodgson P, Li Y (2014) Effects of alloying elements on the corrosion behavior and biocompatibility of biodegradable magnesium alloys: a review. J Mater Chem B 2: 1912–1933. 10.1039/c3tb21746a 32261628

[17] Song Y, Han E-H, Shan D, Yim CD, You BS (2012) The effect of Zn concentration on the corrosion behavior of Mg–xZn alloys. Corrosion Science 65: 322–330.

[18] Walker J, Shadanbaz S, Woodfield TB, Staiger MP, Dias GJ (2014) Magnesium biomaterials for orthopedic application: a review from a biological perspective. J Biomed Mater Res B Appl Biomater 102: 1316–1331. 10.1002/jbm.b.33113 24458999

[19] Yu K, Chen L, Zhao J, Li S, Dai Y, et al (2012) In vitro corrosion behavior and in vivo biodegradation of biomedical beta-Ca3(PO4)2/Mg-Zn composites. Acta Biomater 8: 2845–2855. 10.1016/j.actbio.2012.04.009 22503951

[20] Tang J, Wang J, Xie X, Zhang P, Lai Y, et al (2013) Surface coating reduces degradation rate of magnesium alloy developed for orthopaedic applications. Journal of Orthopaedic Translation 1: 41–48.

[21] Wang J, Tang J, Zhang P, Li Y, Wang J, et al (2012) Surface modification of magnesium alloys developed for bioabsorbable orthopedic implants: a general review. J Biomed Mater Res B Appl Biomater 100: 1691–1701. 10.1002/jbm.b.32707 22566412

[22] Cui W, Beniash E, Gawalt E, Xu Z, Sfeir C (2013) Biomimetic coating of magnesium alloy for enhanced corrosion resistance and calcium phosphate deposition. Acta Biomater 9: 8650–8659. 10.1016/j.actbio.2013.06.031 23816653

[23] Waterman J, Pietak A, Birbilis N, Woodfield T, Dias G, et al (2011) Corrosion resistance of biomimetic calcium phosphate coatings on magnesium due to varying pretreatment time. Materials Science and Engineering: B 176: 1756–1760.

[24] Hiromoto S, Inoue M, Taguchi T, Yamane M, Ohtsu N (2015) In vitro and in vivo biocompatibility and corrosion behaviour of a bioabsorbable magnesium alloy coated with octacalcium phosphate and hydroxyapatite. Acta Biomater 11: 520–530. 10.1016/j.actbio.2014.09.026 25257316

[25] Razavi M, Fathi M, Savabi O, Vashaee D, Tayebi L (2015) In vivo assessments of bioabsorbable AZ91 magnesium implants coated with nanostructured fluoridated hydroxyapatite by MAO/EPD technique for biomedical applications. Mater Sci Eng C Mater Biol Appl 48: 21–27. 10.1016/j.msec.2014.11.020 25579892

[26] Lin X, Tan L, Wang Q, Zhang G, Zhang B, et al (2013) In vivo degradation and tissue compatibility of ZK60 magnesium alloy with micro-arc oxidation coating in a transcortical model. Mater Sci Eng C Mater Biol Appl 33: 3881–3888. 10.1016/j.msec.2013.05.023 23910291

[27] Fischerauer SF, Kraus T, Wu X, Tangl S, Sorantin E, et al (2013) In vivo degradation performance of micro-arc-oxidized magnesium implants: a micro-CT study in rats. Acta Biomater 9: 5411–5420. 10.1016/j.actbio.2012.09.017 23022544

[28] Yu W, Zhao H, Ding Z, Zhang Z, Sun B, et al (2017) In vitro and in vivo evaluation of MgF2 coated AZ31 magnesium alloy porous scaffolds for bone regeneration. Colloids Surf B Biointerfaces 149: 330–340. 10.1016/j.colsurfb.2016.10.037 27792982

[29] Hornberger H, Virtanen S, Boccaccini AR (2012) Biomedical coatings on magnesium alloys—a review. Acta Biomater 8: 2442–2455. 10.1016/j.actbio.2012.04.012 22510401

[30] Sun W, Zhang G, Tan L, Yang K, Ai H (2016) The fluoride coated AZ31B magnesium alloy improves corrosion resistance and stimulates bone formation in rabbit model. Mater Sci Eng C Mater Biol Appl 63: 506–511. 10.1016/j.msec.2016.03.016 27040245

[31] Wu G, Ibrahim JM, Chu PK (2013) Surface design of biodegradable magnesium alloys—A review. Surface and Coatings Technology 233: 2–12.

[32] Wong HM, Yeung KW, Lam KO, Tam V, Chu PK, et al (2010) A biodegradable polymer-based coating to control the performance of magnesium alloy orthopaedic implants. Biomaterials 31: 2084–2096. 10.1016/j.biomaterials.2009.11.111 20031201

[33] Razavi M, Fathi M, Savabi O, Tayebi L, Vashaee D (2018) Improvement of in vitro behavior of an Mg alloy using a nanostructured composite bioceramic coating. J Mater Sci Mater Med 29: 159 10.1007/s10856-018-6170-1 30350229

[34] Heise S, Virtanen S, Boccaccini AR (2016) Tackling Mg alloy corrosion by natural polymer coatings-A review. J Biomed Mater Res A 104: 2628–2641. 10.1002/jbm.a.35776 27159153

[35] Zhou X, OuYang J, Li L, Liu Q, Liu C, et al (2019) In vitro and in vivo anti-corrosion properties and bio-compatibility of 5β-TCP/Mg-3Zn scaffold coated with dopamine-gelatin composite. Surface and Coatings Technology 374: 152–163.

[36] Fu Y, Du L, Wang Q, Liao W, Jin Y, et al (2012) In vitro sustained release of recombinant human bone morphogenetic protein-2 microspheres embedded in thermosensitive hydrogels. Pharmazie 67: 299–303. 22570935

[37] Marie PJ, Debiais F, Hay E (2002) Regulation of human cranial osteoblast phenotype by FGF-2, FGFR-2 and BMP-2 signaling. Histol Histopathol 17: 877–885. 10.14670/HH-17.877 12168799

[38] Xia P, Wang S, Qi Z, Zhang W, Sun Y (2019) BMP-2-releasing gelatin microspheres/PLGA scaffolds for bone repairment of X-ray-radiated rabbit radius defects. Artif Cells Nanomed Biotechnol 47: 1662–1673. 10.1080/21691401.2019.1594852 31032645

[39] Wang H, Boerman OC, Sariibrahimoglu K, Li Y, Jansen JA, et al (2012) Comparison of micro- vs. nanostructured colloidal gelatin gels for sustained delivery of osteogenic proteins: Bone morphogenetic protein-2 and alkaline phosphatase. Biomaterials 33: 8695–8703. 10.1016/j.biomaterials.2012.08.024 22922022

[40] Seyedraoufi ZS, Mirdamadi S (2013) Synthesis, microstructure and mechanical properties of porous Mg—Zn scaffolds. J Mech Behav Biomed Mater 21: 1–8. 10.1016/j.jmbbm.2013.01.023 23454363

[41] (2009) ISO 10993–5:2009(E), Biological evaluation of medical devices—Part 5: Tests for in vitro cytotoxicity. International Organization for Standardization.

[42] Zhang L, Li Q, Liu Z, Wang Y, Zhao M (2019) The protective effects of bone mesenchymal stem cells on paraquat-induced acute lung injury via the muc5b and ERK/MAPK signaling pathways. Am J Transl Res 11: 3707–3721. 31312382 PMC6614636

[43] Chen D, Zhang X, He Y, Lu J, Shen H, et al (2012) Co-culturing mesenchymal stem cells from bone marrow and periosteum enhances osteogenesis and neovascularization of tissue-engineered bone. J Tissue Eng Regen Med 6: 822–832. 10.1002/term.489 22072318

[44] Zhuang H, Han Y, Feng A (2008) Preparation, mechanical properties and in vitro biodegradation of porous magnesium scaffolds. Materials Science and Engineering: C 28: 1462–1466.

[45] Narayan R (2012) ASM Handbook: Materials for Medical Devices: ASM International 136–181 p.

[46] Zhang ZH, Zhang DQ, Zhu LH, Gao LX, Lin T, et al (2017) Performance enhancement of the anti-corrosion coating based on Ce3+-polyaniline–montmorillonite composite/epoxy-ester system. Journal of Coatings Technology and Research 14: 1083–1093.

[47] Cai S, Lei T, Li N, Feng F (2012) Effects of Zn on microstructure, mechanical properties and corrosion behavior of Mg–Zn alloys. Materials Science and Engineering: C 32: 2570–2577.

[48] Witte F, Feyerabend F, Maier P, Fischer J, Stormer M, et al (2007) Biodegradable magnesium-hydroxyapatite metal matrix composites. Biomaterials 28: 2163–2174. 10.1016/j.biomaterials.2006.12.027 17276507

[49] Saidin S, Chevallier P, Abdul Kadir MR, Hermawan H, Mantovani D (2013) Polydopamine as an intermediate layer for silver and hydroxyapatite immobilisation on metallic biomaterials surface. Mater Sci Eng C Mater Biol Appl 33: 4715–4724. 10.1016/j.msec.2013.07.026 24094179

[50] Lin B, Zhong M, Zheng C, Cao L, Wang D, et al (2015) Preparation and characterization of dopamine-induced biomimetic hydroxyapatite coatings on the AZ31 magnesium alloy. Surface and Coatings Technology 281: 82–88.

[51] Li H, Peng F, Wang D, Qiao Y, Xu D, et al (2018) Layered double hydroxide/poly-dopamine composite coating with surface heparinization on Mg alloys: improved anticorrosion, endothelialization and hemocompatibility. Biomater Sci 6: 1846–1858. 10.1039/c8bm00298c 29789824

[52] Poh CK, Shi Z, Lim TY, Neoh KG, Wang W (2010) The effect of VEGF functionalization of titanium on endothelial cells in vitro. Biomaterials 31: 1578–1585. 10.1016/j.biomaterials.2009.11.042 19963265

[53] Zhu LP, Jiang JH, Zhu BK, Xu YY (2011) Immobilization of bovine serum albumin onto porous polyethylene membranes using strongly attached polydopamine as a spacer. Colloids Surf B Biointerfaces 86: 111–118. 10.1016/j.colsurfb.2011.03.027 21497492

[54] Chen Y, Zhao S, Chen M, Zhang W, Mao J, et al (2015) Sandwiched polydopamine (PDA) layer for titanium dioxide (TiO2) coating on magnesium to enhance corrosion protection. Corrosion Science 96: 67–73.

[55] Lee H, Dellatore SM, Miller WM, Messersmith PB (2007) Mussel-inspired surface chemistry for multifunctional coatings. Science 318: 426–430. 10.1126/science.1147241 17947576 PMC2601629

[56] Popescu S, Ungureanu C, Albu AM, Pirvu C (2014) Poly(dopamine) assisted deposition of adherent PPy film on Ti substrate. Progress in Organic Coatings 77: 1890–1900.

[57] Zwingenberger S, Langanke R, Vater C, Lee G, Niederlohmann E, et al (2016) The effect of SDF-1alpha on low dose BMP-2 mediated bone regeneration by release from heparinized mineralized collagen type I matrix scaffolds in a murine critical size bone defect model. J Biomed Mater Res A 104: 2126–2134. 10.1002/jbm.a.35744 27060915

[58] Hughes-Fulford M, Li CF (2011) The role of FGF-2 and BMP-2 in regulation of gene induction, cell proliferation and mineralization. J Orthop Surg Res 6: 8 10.1186/1749-799X-6-8 21306641 PMC3044105

[59] Kim S, Kang Y, Krueger CA, Sen M, Holcomb JB, et al (2012) Sequential delivery of BMP-2 and IGF-1 using a chitosan gel with gelatin microspheres enhances early osteoblastic differentiation. Acta Biomater 8: 1768–1777. 10.1016/j.actbio.2012.01.009 22293583 PMC3314097

[60] Echave MC, Saenz del Burgo L, Pedraz JL, Orive G (2017) Gelatin as Biomaterial for Tissue Engineering. Curr Pharm Des 23: 3567–3584. 10.2174/0929867324666170511123101 28494717

[61] Qi H, Heise S, Zhou J, Schuhladen K, Yang Y, et al (2019) Electrophoretic Deposition of Bioadaptive Drug Delivery Coatings on Magnesium Alloy for Bone Repair. ACS Appl Mater Interfaces 11: 8625–8634. 10.1021/acsami.9b01227 30715842

[62] Yang S, Wang Y, Luo S, Shan C, Geng Y, et al (2019) Building polyphenol and gelatin films as implant coating, evaluating from in vitro and in vivo performances. Colloids Surf B Biointerfaces 181: 549–560. 10.1016/j.colsurfb.2019.05.058 31185447

[63] Kim YH, Son SR, Sarkar SK, Lee BT (2014) The effects of dimethyl 3,3'-dithiobispropionimidate di-hydrochloride cross-linking of collagen and gelatin coating on porous spherical biphasic calcium phosphate granules. J Biomater Appl 29: 386–398. 10.1177/0885328214530483 24733775

[64] Zeng R, Dietzel W, Witte F, Hort N, Blawert C (2008) Progress and Challenge for Magnesium Alloys as Biomaterials. ADVANCED ENGINEERING MATERIALS 10: B3–B14.

[65] Fischer J, Pröfrock D, Hort N, Willumeit R, Feyerabend F (2011) Improved cytotoxicity testing of magnesium materials. Materials Science and Engineering: B 176: 830–834.

[66] Brown A, Zaky S, Ray H Jr., Sfeir C (2015) Porous magnesium/PLGA composite scaffolds for enhanced bone regeneration following tooth extraction. Acta Biomater 11: 543–553. 10.1016/j.actbio.2014.09.008 25234156

[67] Florian L, Michael F, Karl L, Philipp L, Markus R, et al (2007) Methods in Enzymology. 209–225 p.

[68] Oliveira Barud HG, Barud Hda S, Cavicchioli M, do Amaral TS, de Oliveira OB Junior, et al (2015) Preparation and characterization of a bacterial cellulose/silk fibroin sponge scaffold for tissue regeneration. Carbohydr Polym 128: 41–51. 10.1016/j.carbpol.2015.04.007 26005138

[69] Gao C, Wan Y, Yang C, Dai K, Tang T, et al (2010) Preparation and characterization of bacterial cellulose sponge with hierarchical pore structure as tissue engineering scaffold. Journal of Porous Materials 18: 139–145.

[70] Yoshizawa S, Brown A, Barchowsky A, Sfeir C (2014) Magnesium ion stimulation of bone marrow stromal cells enhances osteogenic activity, simulating the effect of magnesium alloy degradation. Acta Biomater 10: 2834–2842. 10.1016/j.actbio.2014.02.002 24512978

[71] Feyerabend F, Witte F, Kammal M, Willumeit R (2006) Unphysiologically high magnesium concentrations support chondrocyte proliferation and redifferentiation. Tissue Eng 12: 3545–3556. 10.1089/ten.2006.12.3545 17518690

[72] Wong HM, Wu S, Chu PK, Cheng SH, Luk KD, et al (2013) Low-modulus Mg/PCL hybrid bone substitute for osteoporotic fracture fixation. Biomaterials 34: 7016–7032. 10.1016/j.biomaterials.2013.05.062 23787111

[73] Vormann J (2003) Magnesium: nutrition and metabolism. Mol Aspects Med 24: 27–37. 10.1016/s0098-2997(02)00089-4 12537987

[74] Tie D, Guan R, Liu H, Cipriano A, Liu Y, et al (2016) An in vivo study on the metabolism and osteogenic activity of bioabsorbable Mg-1Sr alloy. Acta Biomater 29: 455–467. 10.1016/j.actbio.2015.11.014 26577986

[75] Li X, Liu X, Wu S, Yeung KWK, Zheng Y, et al (2016) Design of magnesium alloys with controllable degradation for biomedical implants: From bulk to surface. Acta Biomater 45: 2–30. 10.1016/j.actbio.2016.09.005 27612959

[76] Shadanbaz S, Dias GJ (2012) Calcium phosphate coatings on magnesium alloys for biomedical applications: a review. Acta Biomater 8: 20–30. 10.1016/j.actbio.2011.10.016 22040686

[77] Dziuba D, Meyer-Lindenberg A, Seitz JM, Waizy H, Angrisani N, et al (2013) Long-term in vivo degradation behaviour and biocompatibility of the magnesium alloy ZEK100 for use as a biodegradable bone implant. Acta Biomater 9: 8548–8560. 10.1016/j.actbio.2012.08.028 22922249

[78] Schaller B, Saulacic N, Imwinkelried T, Beck S, Liu EW, et al (2016) In vivo degradation of magnesium plate/screw osteosynthesis implant systems: Soft and hard tissue response in a calvarial model in miniature pigs. J Craniomaxillofac Surg 44: 309–317. 10.1016/j.jcms.2015.12.009 26805919

[79] de Jonge LT, Leeuwenburgh SC, van den Beucken JJ, te Riet J, Daamen WF, et al (2010) The osteogenic effect of electrosprayed nanoscale collagen/calcium phosphate coatings on titanium. Biomaterials 31: 2461–2469. 10.1016/j.biomaterials.2009.11.114 20022365

